# Identification of water use efficiency related genes in ‘Garnem’ almond-peach rootstock using time-course transcriptome analysis

**DOI:** 10.1371/journal.pone.0205493

**Published:** 2018-10-11

**Authors:** Beatriz Bielsa, Seanna Hewitt, Sebastian Reyes-Chin-Wo, Amit Dhingra, María José Rubio-Cabetas

**Affiliations:** 1 Hortofruticulture Department. Agrifood Research and Technology Centre of Aragon (CITA), Zaragoza, Spain; 2 Molecular Plant Sciences, Washington State University, Pullman, Washington, United States of America; 3 Department of Horticulture, Washington State University, Pullman, Washington, United States of America; 4 Genome Centre, University of California Davis, Davis, California, United States of America; Estacion Experimental del Zaidin, SPAIN

## Abstract

Drought is one of the main abiotic stresses with far-reaching ecological and socioeconomic impacts, especially in perennial food crops such as *Prunus*. There is an urgent need to identify drought resilient rootstocks that can adapt to changes in water availability. In this study, we tested the hypothesis that PEG-induced water limitation stress will simulate drought conditions and drought-related genes, including transcription factors (TFs), will be differentially expressed in response to this stress. ‘Garnem’ genotype, an almond × peach hybrid [*P*. *amygdalus* Batsch, syn *P*. *dulcis* (Mill.) x *P*. *persica* (L.) Batsch] was exposed to PEG-6000 solution, and a time-course transcriptome analysis of drought-stressed roots was performed at 0, 2 and 24 h time points post-stress. Transcriptome analysis resulted in the identification of 12,693 unique differentially expressed contigs (DECs) at the 2 h time point, and 7,705 unique DECs at the 24 h time point after initiation of the drought treatment. Interestingly, three drought-induced genes, directly related to water use efficiency (WUE) namely, *ERF023* TF; LRR receptor-like serine/threonine-kinase *ERECTA*; and *NF-YB3* TF, were found induced under stress. The RNAseq results were validated with quantitative RT-PCR analysis of eighteen randomly selected differentially expressed contigs (DECs). Pathway analysis in the present study provides valuable information regarding metabolic events that occur during stress-induced signalling in ‘Garnem’ roots. This information is expected to be useful in understanding the potential mechanisms underlying drought stress responses and drought adaptation strategies in *Prunus* species.

## Introduction

*Prunus* L. is a diverse and economically important genus belonging to the Rosaceae family. It is comprised of approximately 200 species, most of which grow in the temperate zone, although some are found to inhabit tropical and subtropical regions [1]. The economic importance of this fruit tree genus is evident from the diverse uses of its members as a source of food, oil, and timber, and ornamentals [2].

Commercial production of *Prunus* species requires the use of rootstocks, which are derived from several members of the Amygdaloidae family, namely, *P*. *amygdalus* (L.) Batsch, *P*. *persica* (L.) Batsch, *P*. *cerasifera* Ehrh., *P*. *davidiana* (Carr.) Franch, *P*. *mira* (Koehne) Kov. et Kost., *P*. *domestica* L. and *P*. *insititia* L. Over the years, *Prunus* rootstock improvement, via traditional breeding, has been successful in incorporating various genetic traits such as improved water and nutrient uptake, resistance to soil-borne pathogens, and tolerance to environmental stresses [3]. Almond × peach hybrids such as ‘Garnem’, ‘Felinem’ and ‘Monegro’ (which have been derived from the cross between ‘Garfi’ almond × ‘Nemared’ peach) exhibit high vigour, nematode resistance, and adaptability to calcareous soils [4]. With hybrid rootstocks being increasingly derived from crosses between almond × peach and plum genotypes, the next challenge is to combine the tolerance to biotic and abiotic stresses in the new generation of rootstocks [4–7].

Drought is increasingly becoming one of the main abiotic stresses that threatens global agricultural production, particularly in the arid and semi-arid regions around the Mediterranean. Drought-tolerant plants utilize diverse approaches to survive under stress conditions, and it is critical to understand the molecular basis of the various survival mechanisms. Water-limitation stress signals, which are initially perceived within the roots, and then systemically transmitted throughout the entire plant, result in activation of expression of numerous drought-related genes. This induces a cascade of molecular, cellular and biochemical processes including modifications in stomatal movement [8], accumulation of osmolytes [9], and antioxidant signalling [10,11]. The activation of these processes allows for maintenance of cellular homeostasis through lipid and carbohydrate metabolism [12]. Based on current understanding, drought-responsive genes can be classified into two groups depending on their function: (i) Regulatory genes (e.g. transcription factors (TFs), kinases and phosphatases, and enzymes for phytohormones biosynthesis) and, (ii) Effector genes (e.g. chaperones, late embryogenesis abundant (LEA) proteins, enzymes for osmolytes biosynthesis and water channel proteins) [13,14]. Identification of these genes, and their functional, and mechanistic characterization is critical for the improvement of drought tolerance in economically important crops [15]. Over the last decade, different genomic and genetic tools have been used to identify *Prunus*-specific genes involved in drought response. A comparative expression analysis of three peach dehydrin genes provided an insight into their role during drought and cold-induced stress response [16]. In *P*. *scoparia*, several water-deficit resistance genes involved in ABA biosynthesis such as zeaxanthin epoxidase and sugar signalling as starch synthase VI and protein kinase MK5 (*AFC2*), were identified using the cDNA-AFLP technique [17].

The utilization of high-throughput approaches has revolutionized the ability to elucidate drought responses in plants. Several global gene expression studies using microarray platforms have been reported in tomato [18], rice [19] and in other woody plants, such as *P*. *taeda* [20]. Recently, RNA sequencing (RNAseq) technology has made it possible to capture and compare entire transcriptomes of genotypes exposed to different stress conditions at various time points, while providing greater accuracy and sensitivity than other methods [21]. RNAseq analysis has facilitated characterization of responses under both biotic [22] and abiotic stresses, including low temperature in peach [23], early freezing in maize [24] and root hypoxia in *Prunus* rootstock [25]. Two recent studies identified drought-responsive genes under long-term drought exposure in Mongolian almond [26], and in leaf and root tissues of grafted peach trees [27].

In this study, we tested the hypothesis that PEG-induced water limitation stress will simulate drought conditions in the plants and drought-related genes including TFs will be differentially expressed in response to water limitation stress. ‘Garnem’ rootstocks were exposed to drought, which was induced using the polyethylene glycol (PEG) method. The predicted molecular processes involved in drought stress response, which were identified in the roots of ‘Garnem’ are expected to serve as key information for subsequent investigations related to improvement of water use efficiency (WUE) and thus, drought tolerance in *Prunus*.

## Materials and methods

### Plant material and growth conditions

A total of 20 clonally propagated one-year-old plants from the drought tolerant almond-peach hybrid [*P*. *amygdalus* Batsch, syn *P*. *dulcis* (Mill.) x *P*. *persica* (L.) Batsch], ‘Garnem’, were used for the experiment. The plants were acquired from Agromillora Iberia S.L. nursery (Barcelona, Spain). Prior to the drought experiment, the plants were placed in 5-cm diameter pots with a mix of peat, 30% coconut fiber and 20% sand and maintained in a greenhouse at CITA facilities in Zaragoza, Spain (41°43’28.6”N, 0°48’31.1”W). Plants were watered three times a week and fertilized monthly with 15:9:10 N:P:K + 0.2% MgO (Nitricol). Greenhouse temperatures during the growth period ranged from 28 °C to 18 °C (day / night), with a 12 h day / 8 h night photoperiod (S1 Fig).

### Stress conditions and treatment

The plants were divided into control (n = 12 plants) and treatment groups (n = 8 plants). The control plants were maintained under optimal watering conditions, until field capacity, in their 5-cm diameter pots with the initial substrate during the experiment. In this study, drought stress conditions were simulated by providing osmotic stress to the treatment group of plants. The roots of the plants to be treated were placed in a dialysis membrane containing a peat moss substrate and then were submerged in a Polyethylene glycol, PEG-6000, solution (Sigma-Aldrich, Co. St. Luis, MO, USA) (500 g l^-1^), corresponding to an osmotic pressure of -2,68 MPa as per the Michel and Kaufmann equation [28] (S1 Fig). As the plants acclimatized to the PEG solution, the plant osmotic potential was controlled using a Scholander-type pressure chamber (Soil Moisture Equipment Corp. Santa Barbara, CA, USA) [29] until day 7 (S1 Table). The 0-hour time point for the experiment began at the termination of the 7-day acclimation period. Root samples were harvested at 0 h (four control plants), 2 h (four each of control and treatment plants) and 24 h (four each of control and treatment plants) and flash frozen in liquid nitrogen prior to being transferred to storage at -80 °C for subsequent RNA extraction.

### Plant water status

Leaf Water Potential (LWP) was measured in duplicate for each plant (two leaves, each leaf as one biological replicate) using a Scholander-type pressure chamber (Soil Moisture Equipment Corp. Santa Barbara, CA, USA) [29]. Stomatal conductance (gs) was measured in duplicate for each plant with a Leaf Porometer (Decagon Devices Inc. Pullman, WA, USA). Relative Water Content (RWC) was measured in duplicate (using the same leaves were previously used for LWP measurement) as per previously published method [30]. Briefly, three 1-cm diameter leaf discs were weighed (W) and rehydrated to their turgid weight (TW) by floating them in *petri* plates containing deionized water for 4 h at room temperature. The dry weight (DW) was obtained after 24 h at 80 °C in an oven. RWC was calculated following the Eq (1):

$$RWC\;\%\;=\;\frac{W-DW}{TW-DW}\times 100$$ (1)

Electrolyte Leakage (EL) was calculated from Cell Membrane Stability (CMS) rate. CMS was evaluated in duplicate (two leaves, each leaf as one biological replicate) following previously published protocol [31]. Briefly, three 1-cm diameter leaf discs, previously cleaned twice with deionized water to remove surface-bound electrolytes, were submerged in a 50 ml vial containing 10 ml of deionized water and incubated in the dark for 24 h at room temperature. Conductance was then measured with a conductivity meter (CRISON micro CM 2201, Barcelona, Spain). This measurement was taken for control (C1) and treated (T1) samples. After the measurement, the sample vials were autoclaved for 15 min at 121 °C. When the samples reached room temperature, a second reading was recorded for control (C2) and treated (T2) samples. CMS and EL were calculated according the following formulas (2) and (3):

$$CMS\;\%\;=\;\frac{1-\frac{T1}{T2}}{1-\frac{C1}{C2}}\times 100$$ (2)

EL%=100-CMS% (3)

Each of the parameters described above were measured and recorded at 0, 2 and 24 h for both treatments.

### RNA isolation, cDNA library construction and sequencing

Total RNA was extracted from 0.5 g of root tissue for each time point using the CTAB method described previously [32] with minor modifications [33–35]. Extracted RNA was quantified using a NanoDrop ND-1000 UV-vis spectrophotometer (NanoDrop Technologies, Wilmington, DE, USA). RNA integrity was verified by electrophoresis on a 1% agarose gel. Contaminating genomic DNA was removed using DNase I (TURBO DNA-free Ambion, Life Technologies) per the manufacturer’s instructions. Samples were submitted to Lifesequencing S.L. (Paterna, Valencia, Spain) for RNAseq library preparation and sequencing. A total of 1 μg of cellular RNA (RNA integrity number (RIN) > 7.6) was used for TruSeq RNA library construction (Illumina Inc. San Diego, CA, USA). The mRNA was purified using Oligo(dT) cellulose, and was subsequently fragmented into short pieces. First and second-strand cDNA were synthesized using the fragmented RNA as template. Following purification with the QiaQuick PCR extraction kit (Qiagen, Venlo, The Netherlands), sequencing adapters with identification barcodes were ligated to the fragments in order to distinguish different samples. Fragments with lengths of 200–300 bp were purified by Ampure XP beads (Beckman Coulter, Brea, CA, USA), and selectively amplified via PCR in the final step of the library preparation. A total of 10 libraries were sequenced using an Illumina HiSeq 2000 configuration 100 PE (Illumina Inc. San Diego, CA, USA). The libraries represented the following samples: 0 h control (2 biological replicates), 2 h control and 2 h stressed (2 biological replicates for each treatment), and 24 h control and 24 h stressed (2 biological replicates for each treatment) (S2 Table).

### RNAseq data processing

The Illumina HiSeq generated DNA sequence reads in the 2x100 paired format. The resulting fastq files were imported into the CLC Bio Genomics Workbench 6.0.1(CLC Bio, Aarhus, Denmark) for quality assessment, pre-processing, and assembly. Contigs with less than 2x coverage and / or less than 200 bp in length were filtered out. The original, non-trimmed reads from each individual dataset, were mapped back to the master transcriptome assembly in order to count the number of individual sample reads per contig (S2 Table). The master transcriptome was then exported as a fasta file for downstream functional annotation, and the read counts for each dataset were exported and normalized via Reads Per Kilobase per Million reads (RPKM) method [36]. Finally, RPKM values were compared between drought and stressed treatments, using the 0-hour control as a baseline. Thereafter, the RPKM values used for differential expression analysis were derived from the total read count in a pairwise comparison of treatments (drought and control conditions, and 2 h and 24 h of treatment condition). Only genes with a log10 fold change > 5 and p-value < 0.05 were selected for further analysis (S3 Table).

### Functional annotation, pathway analysis and GO enrichment

Gene Ontology (GO) annotation was conducted using the Blast2GO v. 3.3 [37]. Differentially expressed genes in each of the treatment comparisons were functionally annotated using the Blast2GO functional genomics suite using the default parameters. The ontology annotations were refined using InterPro Scan and expanded using ANNEX. GoSlim was used as an additional annotation step to summarize the resulting information. Furthermore, the KEGG (Kyoto Encyclopedia of Genes and Genomes) pathway analysis was performed to map differentially expressed, annotated transcripts to respective metabolic pathways. GO enrichment was performed using the two-tailed Fisher’s exact test (FDR < 0.05) in order to reveal the over- and under-represented functions during PEG-induced drought stress.

### Quantitative real time PCR validation of DEGs

RNA samples (2,500 ng) from root tissue were reverse transcribed with SuperScript III First-Strand Synthesis System (Invitrogen, Life Technologies, Carlsbad, CA, USA) in a total volume of 21 μl according to the manufacturer’s instructions. Primers were designed for 18 randomly selected differentially expressed genes (DEGs) (S4 Table) using Primer3Plus software [38] with the corresponding transcriptome contig as the query sequence for each primer pair. The amplification of the target regions was evaluated using genomic DNA from ‘Garnem’ genotype as template for quality assurance of the primer pairs [39]. Two microliters of 40X dilution of the synthesized cDNA was used for each amplification reaction in a final volume of 10 μl. qRT-PCR was performed in triplicate for each of the two biological replicates on an Applied Biosystems 7900HT Fast PCR System using iTAQ Universal SYBR Green Supermix (Bio-Rad, Hercules, CA, USA). The amplification conditions consisted of an initial denaturation at 95 °C for 10 min, followed by 40 cycles of 15 s at 95 °C for denaturation, and 1 min at 60 °C for annealing and extension. Amplification was preceded by melting curve analysis. Primers for a translocation elongation factor gene (*TEF2*), designed from the available *P*. *persica TEF2* DNA sequence (Gene Bank accession number TC3544), were used as an internal reference control reaction for the qRT-PCR experiments. Relative expression was quantified using the -2ΔΔCt method [40].

### Statistical analysis

#### Physiological parameters

Statistical analyses were performed with SPSS 21 software package (IBM SPSS Statistics, USA). Before carrying out any statistical analyses, the normality of all the data was assessed using the Kolmogorov-Smirnov test. Data following a normal distribution were subjected to ANOVA to test for significant differences between treatments and among hours. Statistical significance was assessed with Tukey’s test (p ≤ 0.05). In case the assumption of normality was not met at the level of 95% confidence, the data were subjected to non-parametric Kruskal-Wallis’ test (p < 0.05). Following confirmation of normal distribution, statistical differences between treatments for each time point were analysed using the Student’s *t*-test (p ≤ 0.05).

### Availability of supporting data

The raw sequencing data from RNAseq analysis were deposited in the NCBI Sequence Read Archive (SRA) under the accession number SRP134116.

## Results and discussion

### Physiological responses to PEG-induced drought

**Fig 1:**
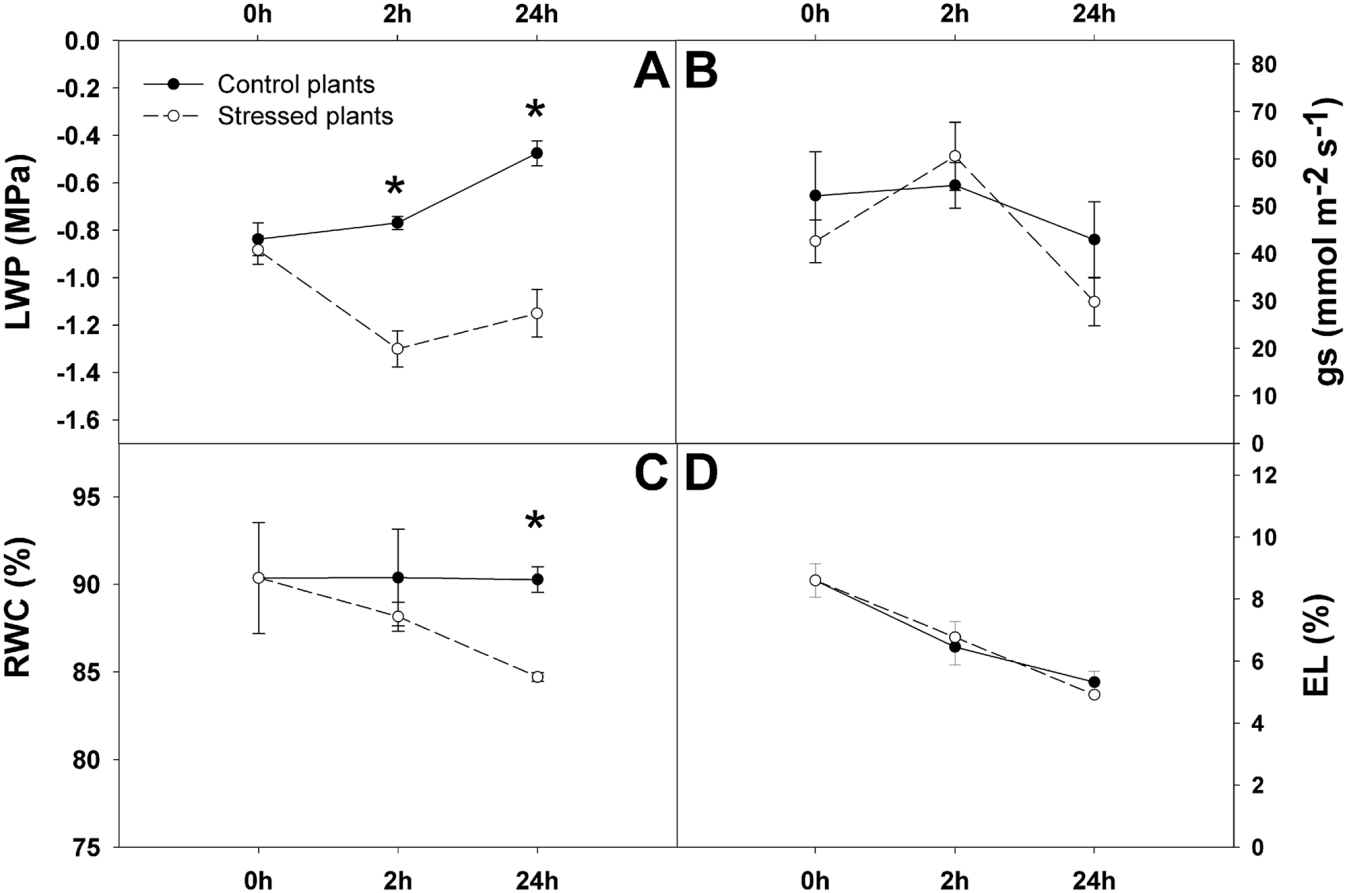
Leaf water potential (LWP) (A), stomatal conductance (gs) (B), relative water content (RWC) (C) and electrolyte leakage (EL) (D) during the drought experiment for control and treated plants of ‘Garnem’. Continuous lines indicate well-watered plants, while dash lines indicate stressed plants. (h = hours. Error bars represent the standard error of the mean. Asterisks represent significant differences (p ≤ 0.05) between treatments (control and stressed) for each time point of the experiment.

The LWP values at the 2 h and 24 h time points reached -0.76 and -0.47 MPa in control plants and -1.30 MPa and -1.15 MPa in PEG-treated ‘Garnem’ plants, respectively (Fig 1A). The LWP was significantly different between the control and treated plants at both 2 h and 24 h time points. The PEG-induced symptoms produced a significant difference in LWP compared to the 0 h time point (Fig 1A).

No significant differences were observed in the stomatal conductance, gs, between control and treated plants at any of the time points (Fig 1B); however, there was a notable decrease in the gs values from 60.57 mmol m^-2^ s^-1^ to 29.85 mmol m^-2^ s^-1^ between 2 h and 24 h time points in the PEG-treated plants (Fig 1B), indicating that the osmotic stress induced stomatal closure. Changes in stomatal conductance from 2 h in the PEG-treated plants are consistent with published literature that reports stomatal closure in drought stress conditions [41,42].

The RWC decreased in the PEG-treated plants throughout the course of the experiment. Significant differences were observed between the control and the treated samples at 24 h, at which the PEG-treated plants reached a minimum RWC value of 84.71% (Fig 1C). As RWC is a good parameter by which cellular water deficit can be extrapolated, these results indicate that the treated ‘Garnem’ samples are in fact experiencing drought stress with respect to the control, particularly at the 24 h timepoint, where the difference is significant. Although RWC decreased at the 24 h time point following PEG treatment, the electrolyte leakage (EL) rate was not significantly affected (1D). Furthermore, EL rates of the PEG-treated plants remained similar to control plants even at low LWP values. While no direct measurement of osmotic potential was conducted, the lack of change in EL indirectly indicates that PEG-mediated osmotic stress may have induced a stress avoidance strategy in the plants mediated by accumulation of solutes leading to an osmotic adjustment under stress conditions [9,42].

The significant changes in physiological parameters associated with stress lend support to the first part of our hypothesis that PEG treatment will induce drought stress in plants. In order to assess the second part of our hypothesis, tissues from plants harvested at two time points that represent the early stages of response to water limiting conditions were subjected to a time-course RNAseq analysis followed by biochemical pathway analysis to identify the underlying metabolic and genetic components.

## Time course RNAseq and biochemical pathway analysis

### Assembly of the time-course RNAseq data and identification of differentially expressed contigs (DECs)

As result of RNAseq analysis, 10 sequenced libraries were obtained. These libraries represented the 0 h, 2 h and 24 h time points of both control and drought-stressed conditions for each one of the two biological replicates of Garnem’ roots studied. An approximate mean Q score of 36 for each library validated the quality of the assay (S2 Table).

In total, approximately 0.42 billion reads, each 100 nucleotides long, were generated, of which 96% (0.4 billion reads) were retained after trimming and filtering low quality reads. Mapping of the original, untrimmed reads from each individual condition and time point back to the master assembly generated 117,356 (79.4%); 140,041 (94.8%); 121,596 (82.3%); 131,251 (88.8%); 138,682 (93.9%) contigs for the 0 h control, 2 h control, 2 h stress, 24 h control and 24 h stress time points, respectively (S2 Table) with a mean contig size of 522 bp. The RPKM values were calculated and used to identify the contigs that were differentially expressed by logFC > 5 in each pairwise comparison (S3 Table).

Four pairwise comparisons of expression values from control and stressed samples at different time points were performed:

1. 2 h Stressed vs. 24 h Stressed (2hS-24hS), which compares changes in transcript expression between PEG treatment time points in order to identify drought-responsive contigs;
2. 2 h Control vs. 2 h Stressed Normalized to 0 h control (2hC-2hSN), which allowed for identification of contigs that were differentially expressed during the first 2 h of drought;
3. 24 h Control vs. 24 h Stressed Normalized to 0 h control (24hC-24hSN), which allowed for identification of genes that were differentially expressed after one day of stress; and
4. 2 h Stressed vs. 24 h Stressed Normalized to 0 h control (2hS-24hSN), which enabled the identification of contigs that changed in expression as a result of PEG addition.

**Fig 2:**
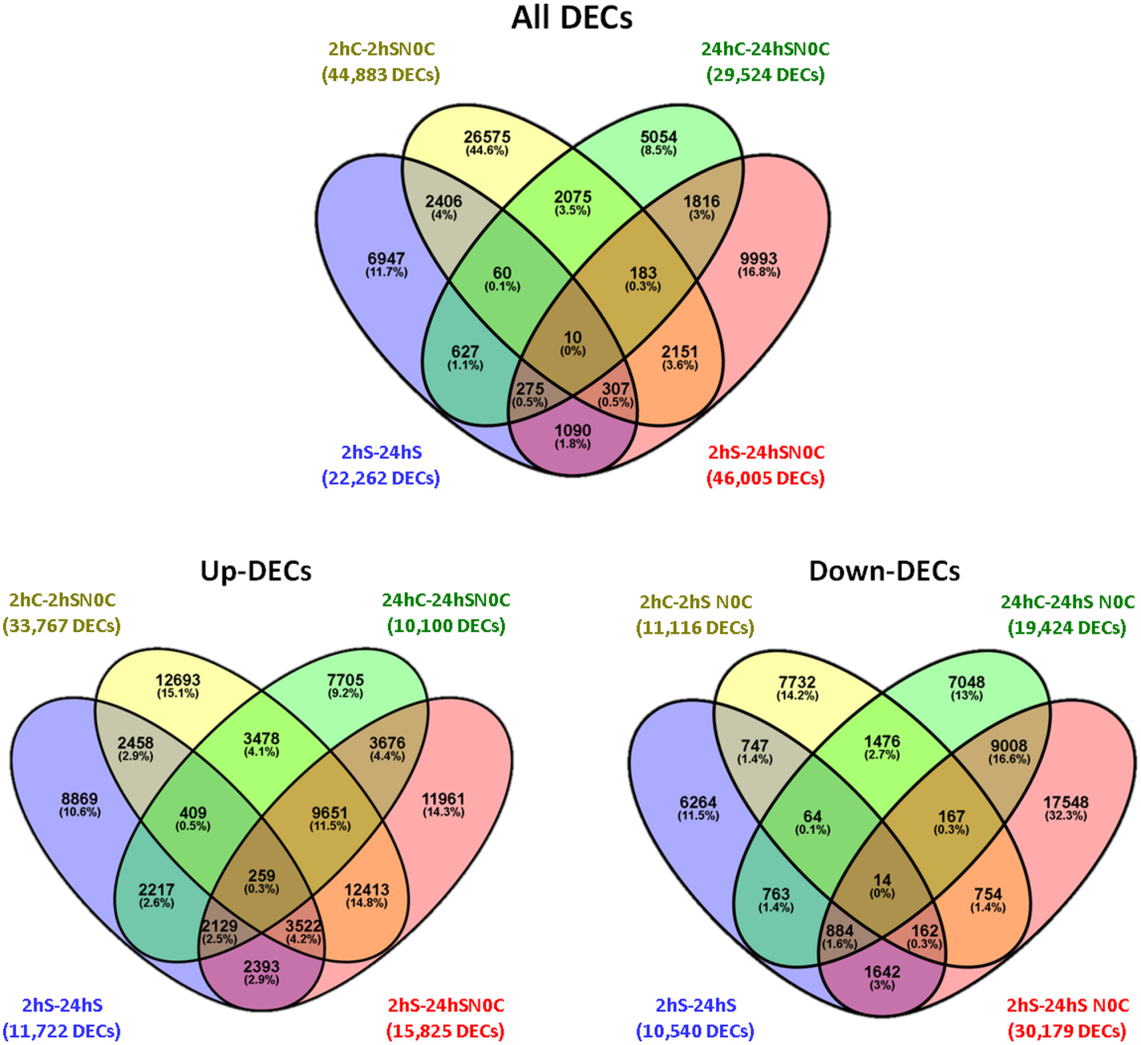
Venn diagrams. Number of DECs (Differentially Expressed Contigs) for the four pairwise comparisons between control and stressed samples collected at different time points: 2 h stressed vs. 24 h stressed (2hS-24hS); 2 h control vs. 2 h stressed normalized to 0 h control (2hC-2hS N0C); 24 h control vs. 24 h stressed normalized to 0 h control (24hC-24hS N0C); and 2 h Stressed vs. 24 h stressed normalized to 0 h control (2hS-24hS N0C).

There was increased transcriptional activity at the 2 h stressed time point in comparison with that of the 24 h stressed time point, with the highest number of differentially expressed contigs (DECs) present exclusively within the 2hC-2hSN pairwise comparison (Fig 2). Notably, only 0.3% of DECs exhibited differential expression across all four pairwise comparisons (Fig 2).

Interestingly, at the 2 h time point of PEG-induced stress, a larger proportion of the genes were upregulated than downregulated. This trend reversed itself at the 24 h time point (Fig 2), perhaps as a result of feedback inhibition of the amplified stress response. These observations indicate that drought-induced transcription occurs primarily in the first few hours of stress as a consequence of the observed physiological adjustments, which may include activation of stress resistance or avoidance mechanisms [9,42]. These observations are consistent with previously published reports on the analysis of transcriptomic response to drought in wheat [43], *Brassica juncea* [44], and in the roots of *Prunus* rootstock under hypoxia conditions [25].

### Functional annotation of the differentially expressed genes (DEGs) and GO term enrichment

**Fig 3:**
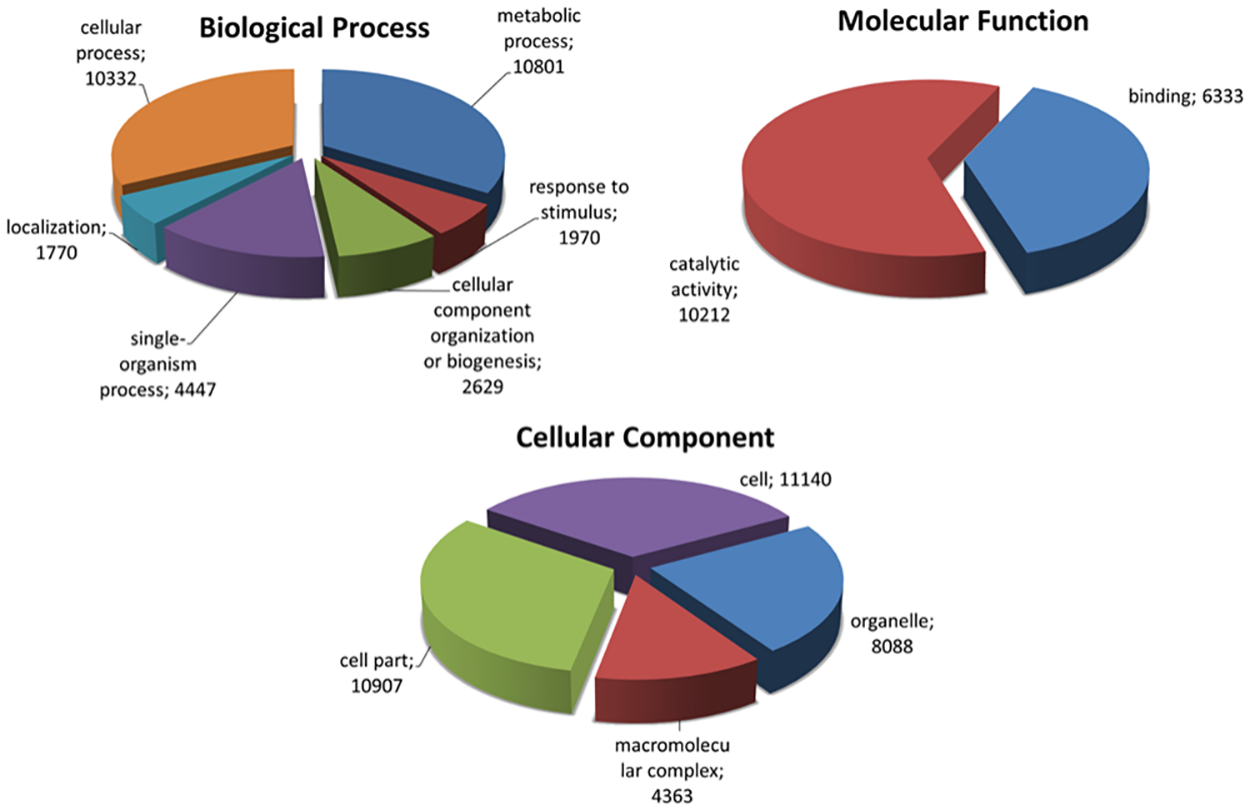
Annotated gene ontology (GO) term distribution at 2-level for the three GO categories after GO-slim analysis.

Of the 83,110 DECs, 49,512 returned positive Blast hits when aligned to the NCBI database with the BLASTX algorithm. The species distribution for the top Blast hits indicated that ‘Garnem’ transcripts had the highest similarity with *P*. *persica* and *P*. *mume*, with 21.6% and 11.1% correspondence, respectively (S2 Fig). Besides peach and Japanese apricot, other *Prunus* species, including *P*. *dulcis*, *P*. *salicina*, *P*. *armeniaca* and *P*. *dulcis × P*. *persica*, were identified as the top hits (S2 Fig). These observations are in agreement with previous studies where a strong homology between various *Prunus* species was reported [26,27]. In total, 26,700 DEGs were annotated and categorized by biological process (BP) (15,870 DEGs), molecular function (MF) (22,595 DEGs) and cellular component (CC) (13,883 DEGs) sets (Fig 3). Due to a lack of sufficient homology with any annotated gene in the *nr* nucleotide database, 1.15% of the DECs were not annotated. They were either classified as proteins of unknown function or as hypothetical proteins. These DEGs may represent important proteins that play a role in drought acclimation, however their function remains to be determined [45,46].

GO enrichment analysis revealed significantly d enriched GO terms at each time point of the PEG-induced drought (see S5 Table). The GO term “growth” was significantly underrepresented at the 2 h stressed treatment (S5 Table). Genes with this ontology are likely associated with numerous cell wall related processes that are activated as a plant adjusts to drought stress. One such process that was observed in ‘Garnem’ was down regulation of *2-dehydro-3-deoxyphosphooctonate aldolase 1* (*KdsA*), and consequent decrease of *3-Deoxy-D-manno-oct-2-ulosonic acid* (kdo) at the 2 h time point. This observation indicates that there is an inhibition of cell wall formation during the early stages of drought response, which is one of the numerous adaptive mechanisms that plants utilize to adjust to new stress conditions [47]. Additional genes enriched under the “growth” ontology term have been implicated in mediating alterations in root growth under stress. Auxin-related genes, such as auxin-binding proteins (ABP4, ABP-T85) and the enzyme AVP1-pyrophosphatase 1 [48] facilitate auxin transport from shoots to roots. Auxin promotes elongation of root cells, thereby enhancing the root system and facilitating increased water uptake from deeper soil layers [49].

### Confirmation of RPKM trends using qRT-PCR

**Fig 4:**
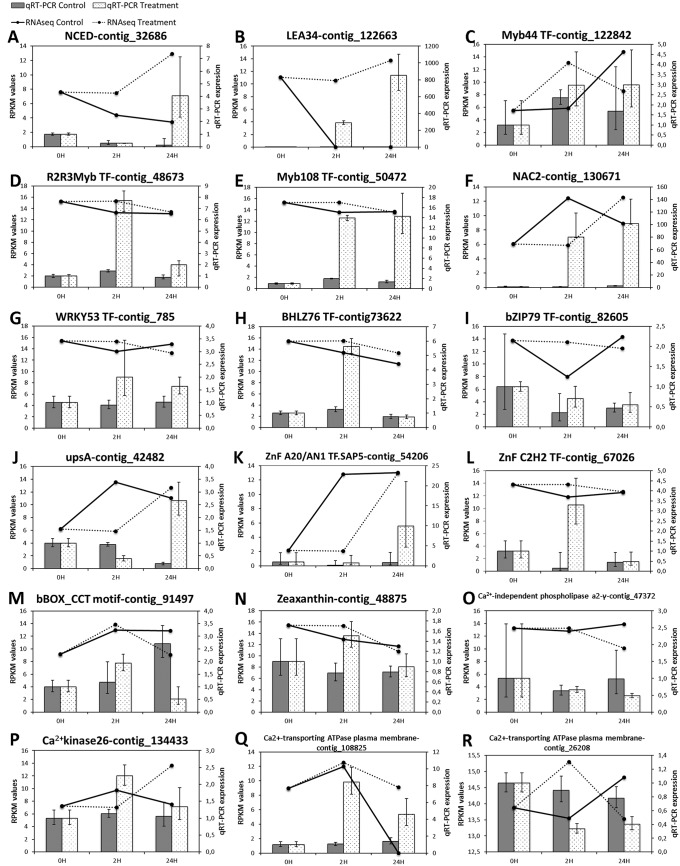
qRT-PCR validation of select genes in control and treated plants. The grey-scale bars represent relative gene expression in control (dark grey) and treated plants (light grey) by qRT-PCR analysis (right y-axis). qRT-PCR data show the average relative expression of two biological samples with three technical replicates each one. Lines represent RPKM values of the transcripts in control (black line) and treated plants (dotted line) by RNAseq (right y-axis). The error bars represent the standard error between replicates in qRT-PCR analysis.

To verify the expression trends determined by RNAseq, qRT -PCR was performed on 18 genes, which represented genes whose RPKM values increased, decreased or remained the same across different time points. The qRT-PCR expression pattern of 16 of the 18 genes (89%) correlated with the RNAseq RPKM values, indicating robustness of the differential gene expression analysis (Fig 4).

### PEG-induced drought activates genes related to stress perception and downstream signalling cascades

Several prior studies have demonstrated that responses to drought are mediated by complex signalling networks that are activated as the plant experiences stress. It is generally considered that a hierarchical sequence of molecular events enables the plants to adapt to stress, which involve stress perception by cell membrane receptors that in turn activate the secondary messengers. As a result, phosphorylation cascades are triggered, resulting in downstream activation of regulatory genes that modulate expression of effector genes of drought stress tolerance [13,49,50].

Based on this paradigm, the annotated DEGs identified in the present study were classified into three major groups: (i) Genes involved in signalling cascades and transcriptional control; (ii) Genes that act as cellular protectors against dehydration-related damage; and (iii) Genes implicated in water and ion uptake and transport [51]. Changes in expression patterns and related discussion for some of the key DEGs annotated under the first category, and important additional mediators of stress signalling is provided in S2 and S3 Appendices sections.

In this study, DEGs annotated as drought-related genes are described in S7 Table. The identity of the drought-related DEGs in ‘Garnem’ rootstocks agrees with previously published studies [26,27, 52].

### Biochemical pathways involved during PEG-induced drought stress

**Fig 5:**
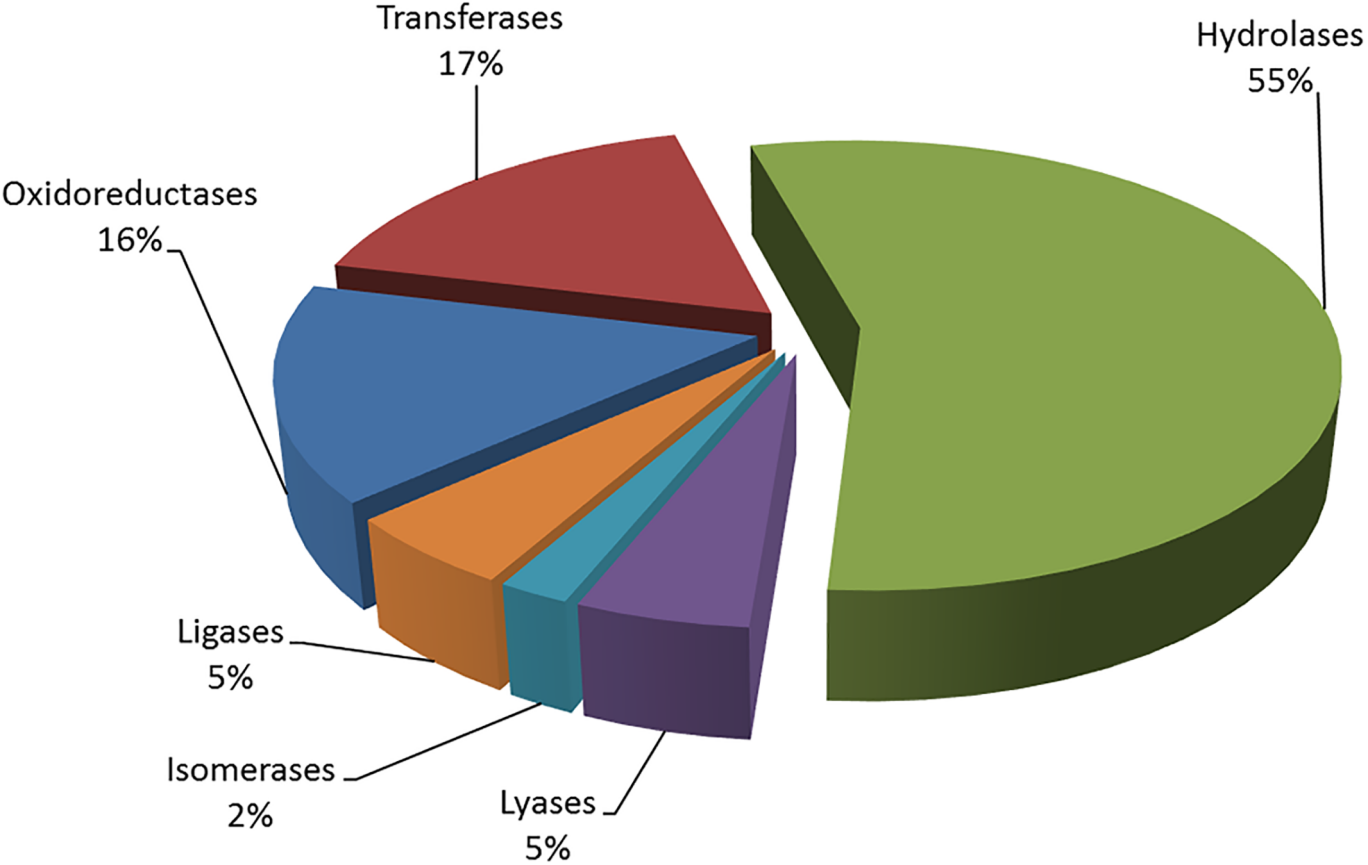
Annotated enzyme distribution of the six main enzyme families in the four DEG pools.

Biochemical pathway analysis was performed to gain a better understanding of the response and adaptation of ‘Garnem’ rootstock to PEG-induced drought conditions by mapping the annotated DEGs to their respective KEGG pathways. It was observed that 718 DEGs identified from the 2hS-24hS comparison mapped to 106 pathways, 2,327 DEGs from the 2hC-2hSN comparison mapped to124 pathways, 2,630 DEGs from the 24hC-24hSN and 3,992 DEGs from the 2hS-24hSN comparison mapped to139 pathways each. Of all the DEGs that mapped to KEGG pathways, 655 were annotated as enzymes, which represented 3 major classes namely Hydrolases (55% of DEGs), Transferases (17%) and Oxidoreductases (16%) (Fig 5, S6 Table).

PEG-induced drought in ‘Garnem’ roots induced several enzymes in purine metabolism (56.42% of DEGs– 51 annotated enzymes), thiamine metabolism (29.56% of DEGs– 6 annotated enzymes), biosynthesis of antibiotics (19.11% of DEGs– 156 annotated enzymes), aminobenzoate degradation (7.62% of DEGs– 6 annotated enzymes), starch and sucrose metabolism (6.44% of DEGs– 32 annotated enzymes) and glycolysis/gluconeogenesis (6.22% of DEGs– 25 annotated enzymes) (S6 Table).

As the most enzyme represented pathway purine metabolism highlights. Metabolites related to purine catabolism contribute to enhance drought tolerance by sensitivity of photosystem reduction, antioxidant protection and ABA metabolism activation [53–55]. It has been demonstrated that Xanthine Dehydrogenase (XDH) acts as a key metabolite in purine catabolism. The suppression and intensification of the XDH activity in rice lines confirmed its role in drought tolerance response by regulating photosystem and reactive oxygen metabolism [55]. In ‘Garnem’ roots, one DEG encoding a Xanthine Hydroxydase (XDH) homolog to AtXDH1 was upregulated at 2 h and downregulated at 24 h time points (S3, S6 and S7 Tables). Then, the XDH enzyme activity might give support antioxidant machinery in ‘Garnem’ rootstock during the first response to drought. On the other side, the accumulation of the intermediary metabolite allantoin functions in drought stress tolerance by influencing in ABA production in *aln* Arabidopsis mutant lines [lines disrupted in allantoinase (ALN) activity], as well as *aah* Arabidopsis mutant lines [disrupted in allantoate amidohydrolase (AAH) activity] submitted to drought conditions [53]. In our results, a DEG encoding the ALN enzyme was upregulated, and a DEG encoding the AAH enzyme was downregulated both at 2 h time point (S3, S6 and S7 Tables). In purine metabolism pathway, ALN acts in a reverse reaction synthetizing allantoate from allantoin, which was catabolized by AAH to obtain ureidoglycine. In ‘Garnem’ roots, these two enzyme activities might have a key impact in the first response to drought. The inhibition activity of the AAH might lead to the synthesis of allantoate which might be catabolized by ALN and then, allowing the accumulation of allantoin metabolite. A deeper study about changes of metabolic activity could confirm the crucial role of these enzymes in ‘Garnem’ rootstock.

Drought-responsive enzymes also mapped to pyruvate metabolism, amino sugar and nucleotide sugar metabolism, pentose phosphate pathway, carbon fixation in photosynthetic organisms, fructose and mannose metabolism, pentose and glucoronate interconversions, carbon fixation pathways in prokaryotes, glyoxylate and dicarboxylate metabolism, galactose metabolism, inositol phosphate metabolism and ascorbate and aldarate metabolism. The involvement of these biochemical pathways has also been observed in previous reports [9,18,56] (S6 and S7 Tables). Additional pathways included lipid metabolism such as glicerolipid metabolism, glicerophospholipid metabolism, fatty acid degradation, fatty acid biosynthesis, sphingolipid metabolism, arachidonic acid metabolism, fatty acid elongation, α-linolenic acid metabolism, steroid hormone biosynthesis of unsaturated fatty acids, ether lipid metabolism, steroid degradation, linoleic acid metabolism, steroid biosynthesis cutin, suberin and wax biosynthesis. These findings agree with previously published studies [12,56–58] (S6 and S7 Tables).

Previous reports have demonstrated that carbohydrate metabolism mediated osmotic adjustment and energy production and preservation are crucial for plant adaptation to water stress [57,59]. Under drought conditions, lipids undergo various changes in their metabolism, which help to maintain cellular homeostasis [12]. Activation of pathways related to carbohydrate and lipid metabolism in ‘Garnem’ transcriptome when exposed to PEG-induced drought indicates that they may be involved in achieving a homeostatic state as has been observed in several previous studies in sweet potato [60], peach [27] and *Lulium multiflorum* [56].

### PEG-induced drought activates genes related to protective mechanisms against dehydration-related damage

Adaptation and tolerance to drought is facilitated by the effector genes that play an important role in regulation of processes involved in cell protective functions. These include heat shock proteins (HSPs) or chaperones, dehydration responsive genes including Late Embryogenesis Abundant (LEA) proteins and dehydrins, osmoprotectants, ROS-responsive genes, transporters, and cell wall modifying enzymes [14].

#### Heat shock proteins (HSPs)

Several HSP encoding genes, which assist in the refolding and stabilization of polypeptides and membranes under stress [61], were represented among the annotated DEGs in ‘Garnem’. In particular, 8 DEGs, annotated as hsp70-Hsp90 organizing-like proteins (HOPs), were found to be upregulated (S3 and S7 Tables). These proteins have previously been implicated in modulation of HSP70/HSP90 interactions and are phosphorylated in roots during drought stress, leading to drought tolerance by binding and stabilizing non-native proteins [62,63]. A group of ‘Garnem’ root HSP chaperone proteins, represented by 33 DEGs, were identified (S7 Table). The functions of many of these chaperones have been previously documented [61]. Therefore, the activity of these HSPs and chaperones as a protein folders, and membrane buffers would be crucial in drought adaptation in ‘Garnem’.

#### Dehydration responsive gene effectors

Multiple chaperone machinery-related proteins as well as 16 DEGs encoding drought-induced chaperonins were identified in the current study (S7 Table). Among these, 7 DEGs annotated as CPN60 and CPN60-like were found to be upregulated at 2 and 24 h time points (S3 Table). This protein acts as folding assistant and peptide assembler, giving support against dehydration damage in cells [64]. Several DEGs encoding LEA proteins and dehydrins, namely, DHN2, COR47, ERD4, LEA D34-like, LEA14 and LEA5 that are known to by strongly induced by dehydration [65], were upregulated in the current dataset at 2 and 24 h time point (S3 and S7 Tables). Their function in chaperone activity and cell membrane protection against water stress, which contributes to enhanced drought tolerance, has been widely reported in *Arabidopsis* [65], P. *persica* [16], *P*. *mume* [66] and in several *Prunus* rootstocks [67]. Other recent transcriptomic studies have found evidence for the role of the LEA and dehydrin proteins in drought response in their respective datasets and plant systems [27,68,69]. Then, all findings suggest that these dehydration responsive gene effectors have a key involvement in drought-adaptive response in ‘Garnem’ rootstock.

#### Drought induced redox homeostasis and antioxidant signalling

Drought stress results in the production of reactive oxygen species (ROS). Excessive accumulation of ROS leads to oxidative stress in plants. Under such conditions, redox homeostasis and antioxidant signalling processes are induced to protect cell membranes and macromolecules [10, 70].

A number of DEGs related to accumulation of antioxidant compounds were induced in roots of ‘Garnem’ under drought (S3 and S7 Tables). These included glutathione S-transferases (GSTs), and 2 DEGs encoding glutathione reductase (GR), and 4 DEGs associated with glutathione peroxidase (GPX), both of which are regulators of oxidative stress response. Ascorbic acid (AsA), which plays a crucial role in plant growth and development as well as human nutrition, is one of the most abundant antioxidants synthesized in plants during drought exposure [70]. In the current data, various DEGs related to AsA biosynthesis, including 2 DEGs encoding a dehydroascorbate reductase (DHAR), 12 DEGs coding for monodehydroascorbate reductases (MDHAR) and MDHAR-like were observed to be differentially expressed during the late stages of drought treatment.

Additionally, a number of DEGs were annotated as antioxidant enzymes, including superoxide dismutases (SODs), peroxidases (POX), ascorbate peroxidases (APX), and catalases (CAT), which were induced under drought conditions. Fruthermore, proteins previously implicated in cell protective and ROS detoxification functions, including ferritins, glutaredoxins, thioredxins and peroxiredoxins, were also found to be differentially expressed in the ‘Garnem’ transcriptome at both 2 and 24 h time points. [11,27,52]. The abundance of transcripts related to ROS scavenging enzymes suggests that under water stress conditions the ROS detoxification system of ‘Garnem’ is particularly effective and may provide improved tolerance to drought.

Of specific interest was a DEG for alterative oxidase (AOX) enzyme that was upregulated. AOX is crucial for limiting ROS production in mitochondria, as well as for maintaining redox homeostasis [71]. Due to its high capacity for alleviating oxidative stress, the AOX enzyme has been proposed as a marker for breeding drought tolerant plant varieties [68].

#### Osmoprotectant biosynthesis genes

Osmoprotectants provide tolerance to drought by encasing cellular structures [9,72]. Furthermore, under drought conditions, the accumulation of compatible osmolytes or osmoprotectants aids in maintaining cellular water content and turgor. In ‘Garnem’ roots, different genes play a role as osmoprotectans were identified, suggesting their implication in the drought response in ‘Garnem’ rootstock. For example, genes related to the biosynthesis of sugars and sugar alcohols, namely trehalose and mannitol, were observed to be differentially expressed under drought stress in ‘Garnem’ at 2 and 24 h time points. In addition, fifteen DEGs were annotated as alpha, alpha-trehalose-phosphate synthases (TPS), 2 DEGs as trehalose-phosphate phosphatases (TPP), 7 DEGs as probable TPPs, and 7 DEGs as probable mannitol dehydrogenases (S7 Table) [9,15,73]. DEGs involved in the synthesis of sucrose and inositol, two other osmoprotectants, were found to be upregulated (S3 and S7 Tables). Additionally, upregulation of DEGs encoding galactinol synthase (GolS1), an enzyme in the raffinose family of oligossacharides (RFOs), whose role in drought-stress response has been demonstrated in *P*.*trichocarpa* [74] was observed in the ‘Garnem’ transcriptome data (S3 and S7 Tables).

Furthermore, 13 DEGs coding for enzymes involved in proline accumulation were observed to be upregulated (S3 and S7 Tables). Increased proline is associated with changes in leaf water potential (LWP) following abiotic stress exposure [52,75,76]. As seen in numerous plant species, proline-based adaptation to drought stress may also be operative during drought stress in ‘Garnem’ [9]. The osmoprotectant-related transcripts induced in ‘Garnem’ are similar to genes previously reported in *P*. *euphratica* subjected to water stress [52]. This indicates the importance of these genes in facilitating osmotic adjustment under drought exposure in ‘Garnem’ rootstock.

#### Protection of cell wall

Water deprivation triggers changes in cell wall composition to minimize water loss. The cell’s first barrier against dehydration, the cuticle, is composed of cutin and wax, hydrophobic substances that limit the amount of water that can exit the cell [77]. Multiple DEGs related to the biosynthesis of cutin and wax accumulation were upregulated in ‘Garnem’ roots exposed to PEG-induced drought (S3 and S7 Tables). These genes included, 3-ketoacyl-synthase-like (KSC), 3-oxoacyl-[acyl-carrier-] synthase chloroplastic-like, and ECERIFERUM enzymes [77,78]. Additional genes associated with cell wall strengthening components, including xyloglucan metabolizing enzymes (xyloglucan endotransglucosylase hydrolases (XTHs), a xyloglucan 6-xylosyltransferase (XXT) and α-xylosidases), expansins, chitinases and enzymes related to biosynthesis of pectin (pectinestearases), and cellulose (COBRA and cellulose synthase enzymes) were also observed to be induced under drought. All of these enzymes participate in controlling cell strength and extension via modification of root structure thereby contributing to drought stress adaptation [68,79,80].

Transport of metal ions, lipids, sugars and other solutes, and water across the vacuolar and plasma membranes is crucial for maintaining all the functional processes especially under abiotic stress conditions. While these results have been seen in other species [68], this is the first time they have been observed in ‘Garnem’ roots. Many of these transporter genes involved in ion movement and water uptake were seen to be induced under drought stress in ‘Garnem’ roots (S7 Table). Description of DEGs and discussion related to the observations are provided in S4 Appendix section.

### Stomatal movement and water use efficiency (WUE) modulate PEG-induced drought response

ABA-induced stomatal closure reduces water loss, stimulates leaf senescence, downregulates plant growth, and induces biosynthesis of protective substances [81]. Regulation of stomatal closure via ABA accumulation results in reduced transpiration, thereby improving water use efficiency (WUE) of the plant [82]. WUE has been recognized as the most important indicator of plant drought adaptation and tolerance [83,84].

Under drought conditions, ABA regulates changes in turgor of guard cells, thereby modulating stomatal movements and flux of CO_2_ and water in plants [8]. The accumulation of ABA is sensed by the PYR1/PYL/CAR receptors, which inhibit the PP2C phosphatase-mediated dephosphorylation of SnRK2 kinases, such as SNF1 [85]. As a result, phosphorylated SnRK2 kinases activate ABRE-binding transcription factors (ABF), which in turn result in the induction of ABA-responsive genes, resulting in stomatal closure [69,86,87].

In PEG-stressed ‘Garnem’ roots, genes representing PYL2-like and PYL8-like ABA receptors were observed to be upregulated (S3 and S7 Tables). However, PYL4-like receptors, which are recognized by Jasmonic Acid [88], were downregulated (S3 and S7 Tables). Most of the DEGs coding for PP2C phosphatases were seen to be downregulated at the 24 h time point, indicating inhibition by PYR1/PYL/RCAR receptors. In this dataset, SNF1-related protein kinases were observed to be upregulated (S3 and S7 Tables). It has been suggested that some members of this gene family may be involved in the regulation of ABA-induced stomatal movement [89,90].

Several contigs were annotated as SnRK2 substrates, which were predicted to be localized in the guard cell membranes. These included the K^+^ channel, KAT1-like and the S-type anion channel SLAH-2-like (homologous to SLAH3). The former was observed to be downregulated at the 24 h time point, while the latter was upregulated at the 2 h time point (S3 and S7 Tables). These observations, which is the first time they have been observed in ‘Garnem’ roots, are similar to a previous report in *Arabidopsis*, where SLAH3 impairs the inward-rectifying K^+^ channel KAT1 in guard cells, thereby keeping the stomata closed during drought stress conditions [91]. In addition to the above-mentioned genes, ABI5, an ARM repeat protein interacting with ABF2 (ARIA), was induced during drought. This protein positively regulates ABA response in *Arabidopsis* by interacting with the ABF2 protein [92].

Furthermore, in the ‘Garnem’ transcriptome data, other DEGs implicated in ABA-mediated stomatal closure were identified. These include WRKY TFs and NAC TFs, which were represented by a number of DEGs that were overexpressed during drought in ‘Garnem’ roots (S7 Table). These TFs may act as positive or negative regulators of stomatal movements via ABA signalling [93,94,95]. There were several additional ABA-related DEGs were indicated to be involved in mediating PEG-induced drought response, which are described and discussed in S5 Appendix section. It is important to note that the pathway modelling analysis was conducted on transcriptome data obtained from root tissues. The differential expression of genes only represents transcript behavior and the correlative change on physiological processes is expected in the correct spatial (tissue) context only. The observed differential expression of genes related to stomatal closure or photosynthetic processes is correlated to the physiological measurements at the 24 h time point, at which stomatal conductance decreased significantly in the PEG-mediated osmotically stressed plants. This suggests that ‘Garnem’ most likely adapts to stress conditions by reducing transpiration via stomatal closure, thereby reducing water loss and modulating the photosynthetic processes.

Additional photosynthesis-related genes that were found to be differentially expressed are listed in S3 and S7 Tables. Previous studies have suggested that the observed changes in photosynthetic proteins could be due in part to compensation of photosynthetic electron transport or enzyme activity, which would maintain a partially open state of stomata during drought, leading in turn to maintenance of normal root growth [52].

In addition, contigs annotated as hexokinase I, which are involved in sugar signalling and metabolism, were found differentially expressed in the current dataset (S7 Table). It has been shown that Hexokinase I regulates stomatal closure [96], thereby reducing stomatal conductance and transpiration, resulting in improved WUE. These proteins may have a similar role in the regulation of stomatal movement during drought adaptation in ‘Garnem’.

Three key DEGs that play a role in enhancing WUE were found to be upregulated at the 2 h time point. These included, *contig_78795*, annotated as *ERF023* TF, *contig_134330* annotated as LRR receptor-like serine/threonine-kinase ERECTA, and *contig_128543* annotated as *NF-YB3* TF (S7 Table).

*PpERF023* (*ppa026139m*) is homologous to the *AtHARDY* gene (*At2g36450*), and AP2/ERF-like TF. A previous study in rice demonstrated that *AtHARDY* improves WUE by enhancing assimilation of photosynthates and decreasing transpiration, thereby resulting in improved drought response [97]. In ‘Garnem’, the HARDY gene may play a role in maintenance of root growth processes that are required for drought adaptation.

The second DEG, *contig_134330*, is homologous to *ppa00847m* (LRR receptor-like serine/threonine-kinase ERECTA isoform X2). Overexpression of *PdERECTA* in transgenic *Arabidopsis* enhanced WUE by eliciting changes in leaf epidermal and mesophyll differentiation, which in turn positively affected growth and accumulation of biomass [98].

The third DEG, *contig_128543* annotated as *NF-YB3-like* TF, which is homologous to *PdNF-YB7*, is a TF that is induced by osmotic stress and ABA. The overexpression of this gene has been shown to promote primary root elongation and increased photosynthesis, thereby conferring increased WUE and drought tolerance in transgenic *Arabidopsis* lines [99]. This finding suggests that *NF-YB3-like* may have a similar function in ‘Garnem’ to those in *Arabidopsis* and could, therefore, potentially increase WUE in *Prunus*.

Based on the previous identification of genes directly related to WUE improvement in rice and *Arabidopsis* [100–102], these three DEGs (*ERF023* TF, *ERECTA* gene and *NF-YB3-like* TF) identified in ‘Garnem’ transcriptome represent promising targets for further characterization in the context of improving drought tolerance in *Prunus*.

## Conclusion

The RNAseq-based biochemical pathway analysis performed in this study represents a comprehensive and temporal analysis of the transcriptomic changes as ‘Garnem’ roots respond to PEG-induced drought conditions. The observed changes in physiological parameters and concomitant changes in expression of genes related to various physiological, biochemical and developmental processes that are known to be involved in response to stress from various studies in multiple plant systems indicates that the PEG-induced method was effective in simulating water-limiting conditions as hypothesized. This provides a set of candidate genes that could be targeted for improved WUE in *Prunus* breeding endeavours.

PEG-induced drought induced the expression of several genes at the 2 h time point, which could be categorized as the primary responders to the water limiting conditions. Some of these key genes included *DREB2B*, the bZIP *TRAB-1-like* and *ALFIN-LIKE 5*. These genes represent TFs, which play a crucial role in drought adaptation by regulating stomatal closure, inducing effector genes and suppressing the expression of negative-effect genes during early drought response. Important among these are the WUE-regulating genes *ERF023* TF, LRR receptor-like serine/threonine-kinase ERECTA, and *NF-YB3* TF. As the plant establishes a homeostatic stage with the new water limiting conditions by 24 h duration, expression of several secondary responder genes is induced. Some of these genes and related functions include osmoprotectants as the enzyme sucrose synthase 7 and enzymes acting in the maintenance of the redox homeostasis such as GSTs and the AOX. The role of several of these key genes in drought response will need to be characterized further, and the information gleaned from such studies is expected to aid in the breeding of drought tolerant *Prunus* species.

## Supporting information

S1 Fig Plant material. (A) Detail of plants from control group. (B) Detail of dialysis membrane in a plant of stressed group. (C and D) Soil humidity differences between a (C) stressed plant after PEG6000 treatment and (D) control plant.
(PDF)

S2 Fig Specie distribution of the first 30 BLAST hits per each contig. (PDF)

S1 Table Physiological monitoring of LWP in PEG-treated plants during both acclimation and drought stress periods. (PDF)

S2 Table Summary of reads from RNA-seq analysis in each generated library and number of genes mapped back to the master transcriptome assembly. (XLSX)

S3 Table List of total differentially expressed contigs (DECs) involved in PEG-treated experiment. (RPKM: Reads Per Kilobase per Million; FC: Fold of change).
(XLSX)

S4 Table Primer list and amplicon sizes for genes in roots of ‘Garnem’ selected for qRT-PCR validation. (XLSX)

S5 Table List of the significantly enriched GO terms for the four pools of differentially expressed genes (DEGs). (XLSX)

S6 Table List of total metabolic pathways for the four pools of differentially expressed genes (DEGs) involved in PEG-treated experiment. (XLSX)

S7 Table List of the total annotated differentially expressed genes (DEGs) involved in PEG-treated experiment. DEG classification is based on its involvement in the drought stress response.
(XLSX)

S1 Appendix Confirmation of RPKM trends using qRT-PCR. (DOCX)

S2 Appendix Genes involved in signalling cascades and transcriptional control. (DOCX)

S3 Appendix Additional mediators of stress signal. (DOCX)

S4 Appendix Genes involved in uptake of water and transport of ions. (DOCX)

S5 Appendix Stomatal movement is modulated by ABA-related genes. (DOCX)
