# Supplementary material for: Identification of water use efficiency related genes in ‘Garnem’ almond-peach rootstock using time-course transcriptome analysis
Source: PLoS One. 2018 Oct 11;13(10):e0205493. doi: 10.1371/journal.pone.0205493 (PMC6181374; doi:10.1371/journal.pone.0205493)
Supplement: S1 Fig — (A) Detail of plants from control group. (B) Detail of dialysis membrane in a plant of stressed group. (C and D) Soil humidity differences between a (C) stressed plant after PEG6000 treatment and (D) control plant. (PDF) [file pone.0205493.s001.pdf]

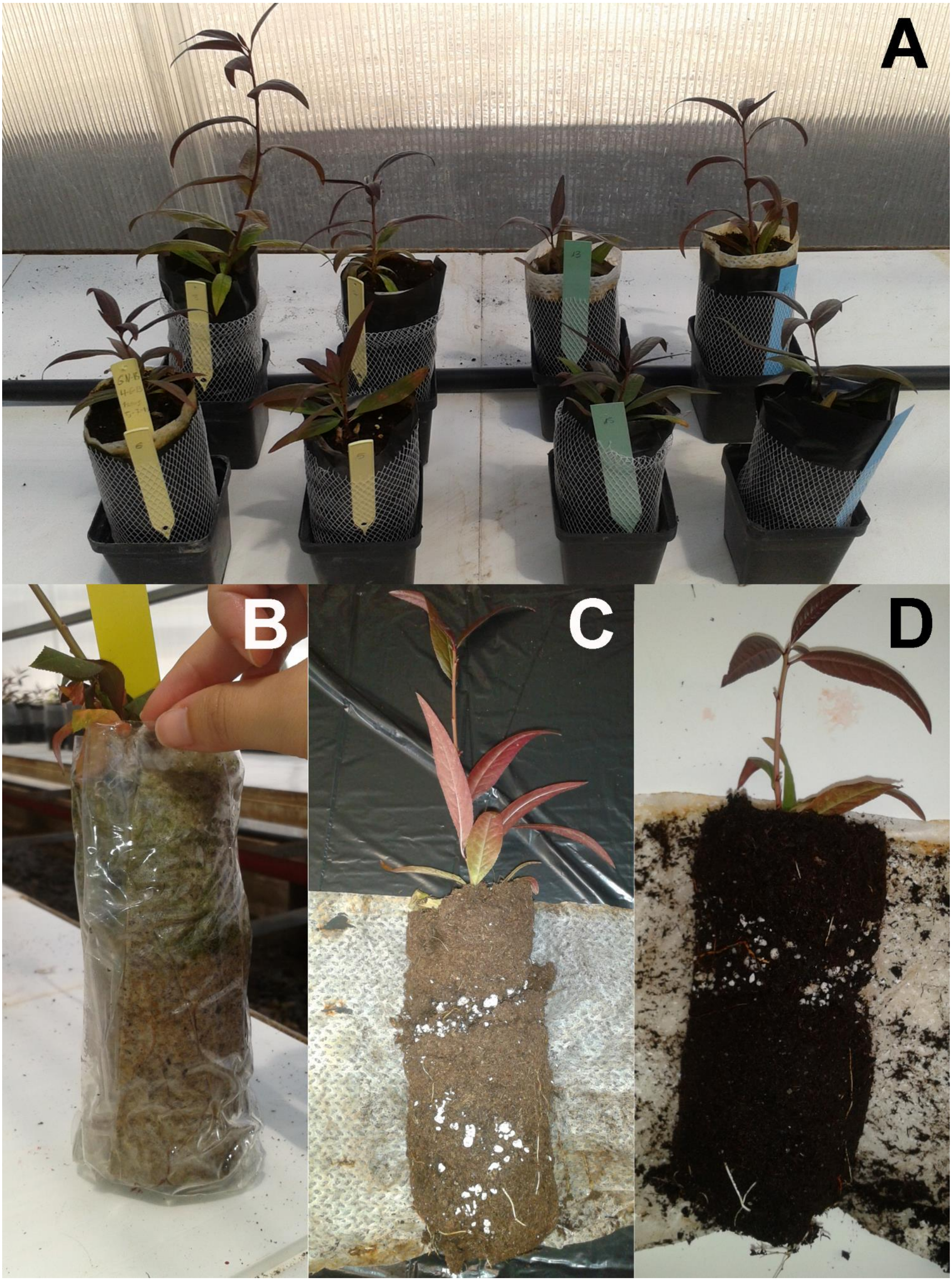

S1 Figure. Plant material. (A) Detail of plants from control group. (B) Detail of dialysis membrane in a plant of stressed group. (C and D) Soil humidity differences between a (C) stressed plant after PEG6000 treatment and (D) control plant.
