# Supplementary figures and images for: Identification of water use efficiency related genes in ‘Garnem’ almond-peach rootstock using time-course transcriptome analysis
Source: PLoS One. 2018 Oct 11;13(10):e0205493. doi: 10.1371/journal.pone.0205493 (PMC6181374; doi:10.1371/journal.pone.0205493)

**S2 Figure. Specie distribution of the first 30 BLAST hits per each contig.**

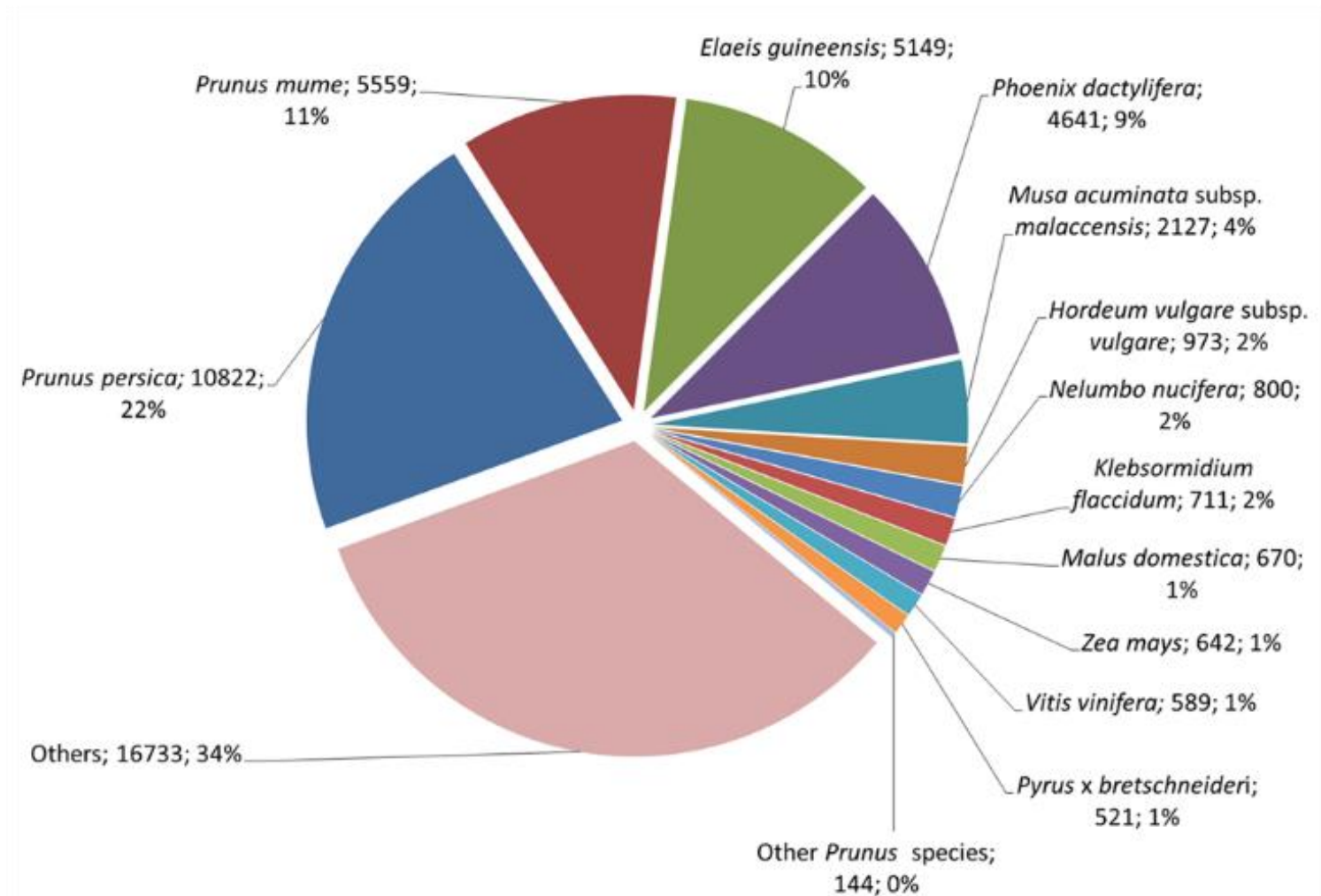

Supplement: S2 Fig — (PDF) [file pone.0205493.s002.pdf]
