# Supplementary material for: Identification of water use efficiency related genes in ‘Garnem’ almond-peach rootstock using time-course transcriptome analysis
Source: PLoS One. 2018 Oct 11;13(10):e0205493. doi: 10.1371/journal.pone.0205493 (PMC6181374; doi:10.1371/journal.pone.0205493)

S1 Table. Physiological monitoring of LWP in PEG-treated plants during both acclimation and drought stress periods.

LWP (kPa)

Acclimation Period

		Monitoring plants	
		Control	Stressed
31-Jul	9 a.m.	-0.700	-0.750
1-Aug	9 a.m.	-0.750	-0.650
2-Aug	9 a.m.	-0.600	-0.500
3-Aug	9 a.m.	-0.850	-0.750
4-Aug	9 a.m.	-0.800	-0.700
5-Aug	9 a.m.	-0.950	-0.750
6-Aug	9 a.m.	-0.800	-0.700

Stress Period

		Ind. Code	control	Stressed	Ind. Code	
7-Aug	0h - 9 a.m.	GN 15_1	-0.9	†	GN 15_9	
		GN 15_2	-0.7	-0.85	GN 15_10	
		GN 15_3	-0.75	-0.8	GN 15_11	
		GN 15_4	-1	-1	GN 15_12	
7-Aug	2h - 11 a.m.	GN 15_5	-0.7	†	GN 15_9	2h - 11 a.m.
		GN 15_6	-0.8	-1.45	GN 15_10	
		GN 15_7	-0.75	-1.2	GN 15_11	
		GN 15_8	-0.825	-1.25	GN 15_12	
8-Aug	24h - 9 a.m.	GN 15_13	-0.5	†	GN 15_17	24h - 9 a.m.
		GN 15_14	-0.35	†	GN 15_18	
		GN 15_15	-0.45	-1.25	GN 15_19	
		GN 15_16	-0.6	-1.05	GN 15_20	

Means for the Stress Period

			Control		Stressed	
			Average	SE	Average	SE
7-Aug	0h - 9 a.m.	**0h**	-0.838	0.069	-0.883	0.060
7-Aug	2h - 11 a.m.	**2h**	-0.769	0.028	-1.300	0.076
8-Aug	24h - 9 a.m.	**24h**	-0.475	0.052	-1.150	0.100

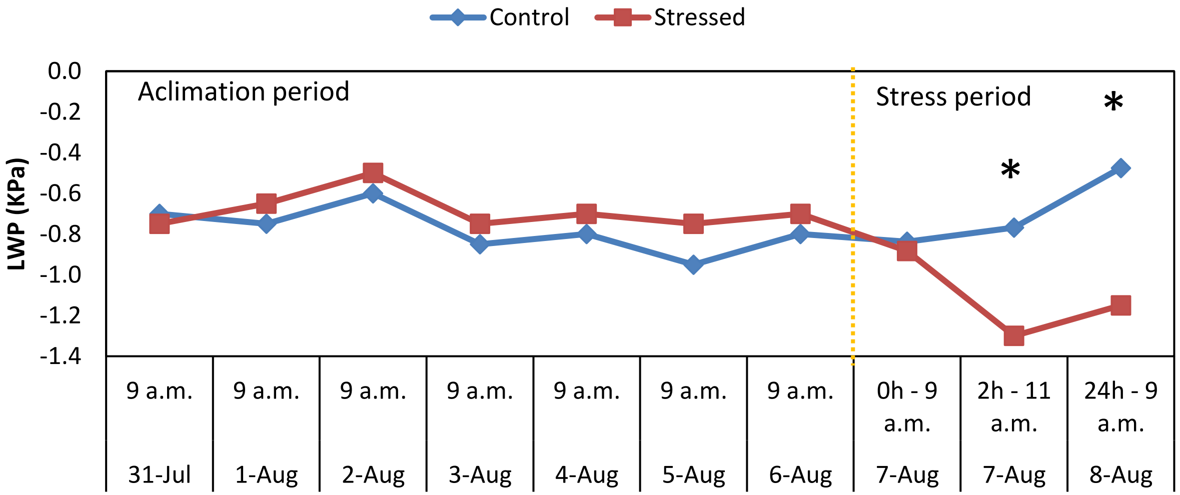

Supplement: S1 Table — (PDF) [file pone.0205493.s003.pdf]
