# Supplementary material for: Identification of water use efficiency related genes in ‘Garnem’ almond-peach rootstock using time-course transcriptome analysis
Source: PLoS One. 2018 Oct 11;13(10):e0205493. doi: 10.1371/journal.pone.0205493 (PMC6181374; doi:10.1371/journal.pone.0205493)
Supplement: S1 Appendix — (DOCX) [file pone.0205493.s010.docx]

# S1.Appendix Sections

**Identification of water use efficiency related genes in ‘Garnem’ almond-peach rootstock using time-course transcriptome analysis**

Beatriz Bielsa^1¶^, Seanna Hewitt^2, 3¶^, Sebastian Reyes-Chin-Wo^4^, Amit Dhingra^2, 3*^, María José Rubio-Cabetas^1*^

**^*^Corresponding authors:**

E-mail: adhingra@wsu.edu (AD)

E-mail: mjrubioc@cita-aragon.es (MJR-C)

## S1. Confirmation of RPKM trends using qRT-PCR

The expression profile of two genes, namely Myb 108 TF and Ca^2+^ Kinase 26, differed between the RNAseq and qRT-PCR methods (Fig 4E and P). This could be due to the targeting of different alleles or primers binding to other targets in the transcriptome.
