# Supplementary material for: Identification of water use efficiency related genes in ‘Garnem’ almond-peach rootstock using time-course transcriptome analysis
Source: PLoS One. 2018 Oct 11;13(10):e0205493. doi: 10.1371/journal.pone.0205493 (PMC6181374; doi:10.1371/journal.pone.0205493)
Supplement: S2 Appendix — (DOCX) [file pone.0205493.s011.docx]

# S2-Appendix Sections

**Identification of water use efficiency related genes in ‘Garnem’ almond-peach rootstock using time-course transcriptome analysis**

Beatriz Bielsa^1¶^, Seanna Hewitt^2, 3¶^, Sebastian Reyes-Chin-Wo^4^, Amit Dhingra^2, 3*^, María José Rubio-Cabetas^1*^

**^*^Corresponding authors:**

E-mail: adhingra@wsu.edu (AD)

E-mail: mjrubioc@cita-aragon.es (MJR-C)

## S2. Genes involved in signalling cascades and transcriptional control

**Signal perception.** **Receptor-like kinases.** At the cell membrane, perception of drought conditions is initiated by receptor-like protein kinases (RLKs), two-component histidine kinases, and G-protein-associated receptors [1].

A total of 595 DEGs encoding RLKs were identified that have been implicated in regulation of abiotic stress response, modulation of disease resistance, and signal transduction [2]. Among these DEGs were included chitin elicitor receptor kinases (CERKs), cysteine-rich receptor kinases (CRKs), receptor-like kinases, histidine kinases (HKs), probable leucine-rich repeat receptor kinases (LRR-RLKs), probable L-type lectin-domain containing receptor kinases (LecRKs), serine/threonine-protein kinases (SnRKs), somatic embryogenesis receptor kinases (SERKs), receptor-like cytosolic serine threonine-kinases, wall-associated receptor kinase-like (WAKs), G-type lectin S-receptor-like serine threonine-kinase (GsSRKs), and strubbelig-receptor family kinases (SRFs). S7 Table provides details regarding the sequence of these genes.

**Secondary messengers.** Initial transduction of the drought stress signal via RLKs is followed by activation of secondary messengers, such as calcium (Ca^2+^), reactive oxygen species (ROS), and inositol phosphates [3]. Ca^2+^ is responsible for coordination and synchronization of diverse stimuli originating from cellular stress responses [3]. Calcium signalling leads to a rapid increase of cytosolic Ca^2+^ in plant cells [4], a response controlled by Ca^2+^ sensors, pumps, transporters and Ca^2+^ channels [5]. Ca^2+^ oscillations are involved in regulation of a number of drought response-related physiological processes, such as stomatal closure [4] and ROS regulation [6]. Several studies have discussed the role of Ca^2+^-dependent protein kinases (CDPKs) in drought tolerance and adaptation, an observation that is further supported by this study. CPK8 and CPK10, for example, enhance tolerance to drought by regulation of stomatal movement in *Arabidopsis* via interaction with CATALASE3 (CAT3) and HSP1 (Heat Shock Protein 1), respectively [7,8]. In the present study, 346 DEGs involved in Ca^2+^ signalling were identified, including several calmodulin coding genes, Ca^2+^ and Ca^2+^- and calmodulin-dependent serine threonine- kinase-like protein encoding genes, Ca^2+^- and calmodulin-dependent kinases, Ca^2+^ uniporter mitochondrial proteins, and various Ca^2+^-binding proteins families including Ca^2+^-binding EF-hand family proteins, calcineurin B-like proteins (CBLs), CDPKs including CPK8 and CPK10, different Ca^2+^-transporting ATPases, and the ER Ca^2+^-binding chaperones calnexin and calreticulin (S3 and S7 Tables). These findings are consistent with the results in other plant systems and provide support for the involvement of Ca^2+^ in response to drought stress in ‘Garnem’ roots [5,6]. Notably, changes in Ca^2+^ levels are also involved in activation of phospholipases C and D, thereby triggering lipid signalling pathways [6]. Three DEGs encoding a Ca^2+^-dependent lipid-binding family protein (CaLB) were identified in this study (S7 Table). This finding suggests that CaLB protein may be involved in binding phospholipase D variants and, consequently, in the activation of lipid signalling-mediated drought adaptation [9].

**Phosphorylation-mediated signal propagation.** Calcium-activated kinases and phosphatases transduce the drought stress signals via a series of protein phosphorylation cascades, including the mitogen-activated protein kinases (MAPKs) and SnRK2 kinases [3,10]. MAPK cascades, which consist of three, interlinked protein kinases [11,12], have been identified in drought response and implicated in crosstalk with both ABA-dependent and ABA-independent signalling pathways [4]. We identified contigs annotated as MAPKs, MAP2Ks and MAP3Ks to be upregulated at the 2 h or 24 h stage (S3 and S7 Tables), indicating that MAPK modulated cascade may be involved in stress signalling in ‘Garnem’. It has previously been suggested that the 14-3-3 proteins may bind and regulate the activity of MAPK phosphatases (MKPs), which play a crucial role in stress responses in wheat [11]. The large amount of upregulated DEGs encoding 14-3-3 protein isoforms in ‘Garnem’ suggests that these proteins may have a similar, important stress responsive role in *Prunus* (S3 and S7 Tables).

**Osmoprotectants control stomatal movements in coordination with ABA-mediated signalling.** Two DEGs that were annotated as probable trehalase (TRE) in ‘Garnem’ transcriptome were observed to be upregulated under drought (S3 and S7 Tables). The overexpression of TRE, which hydrolyzes trehalose into glucose, has been shown to improve drought tolerance by regulating stomatal opening via ABA-dependent signalling [69].

# References

1. Xiong L, Zhu J-K. Abiotic stress signal transduction in plants: Molecular and genetic perspectives. Physiol Plant. 2001;112: 152–166. doi:10.1034/j.1399-3054.2001.1120202.x

2. Ye Y, Ding Y, Jiang Q, Wang F, Sun J, Zhu C. The role of receptor-like protein kinases (*RLKs*) in abiotic stress response in plants. Plant Cell Rep. Springer Berlin Heidelberg; 2017;36: 235–242. doi:10.1007/s00299-016-2084-x

3. Mahajan S, Tuteja N. Cold, salinity and drought stresses: an overview. Arch Biochem Biophys. 2005;444: 139–58. doi:10.1016/j.abb.2005.10.018

4. Roychoudhury A, Paul S, Basu S. Cross-talk between abscisic acid-dependent and abscisic acid-independent pathways during abiotic stress. Plant Cell Rep. 2013;32: 985–1006. doi:10.1007/s00299-013-1414-5

5. Boudsocq M, Sheen J. Stress Signaling II: Calcium Sensing and Signaling. In: Pareek A, Sopory SK, Bohnert HJ, editors. Abiotic Stress Adaptation in Plants: Physiological, Molecular and Genomic Foundation. Dordrecht: Springer Netherlands; 2010. pp. 75–90. doi:10.1007/978-90-481-3112-9

6. Wilkins K, Matthus E, Swarbreck S, Davies J. Calcium-Mediated Abiotic Stress Signaling in Roots. Front Plant Sci. 2016;7: 1–17. doi:10.3389/fpls.2016.01296

7. Zou J-J, Li X-D, Ratnasekera D, Wang C, Liu W-X, Song L-F, et al. Arabidopsis CALCIUM-DEPENDENT PROTEIN KINASE8 and CATALASE3 Function in Abscisic Acid-Mediated Signaling and H_2_O_2_ Homeostasis in Stomatal Guard Cells under Drought Stress. Plant Cell. 2015;27: 1445–60. doi:10.1105/tpc.15.00144

8. Zou J-J, Wei F-J, Wang C, Wu J-J, Ratnasekera D, Liu W-X, et al. Arabidopsis calcium-dependent protein kinase CPK10 functions in abscisic acid- and Ca^2+^-mediated stomatal regulation in response to drought stress. Plant Physiol. 2010;154: 1232–43. doi:10.1104/pp.110.157545

9. Frank W, Munnik T, Kerkmann K, Salamini F, Bartels D. Water deficit triggers phospholipase D activity in the resurrection plant Craterostigma plantagineum. Plant Cell. 2000;12: 111–123.

10. Wang L, Jin X, Li Q, Wang X, Li Z, Wu X. Comparative Proteomics Reveals that Phosphorylation of β-Carbonic Anhydrase 1 Might be Important for Adaptation to Drought Stress in *Brassica napus*. Nat Publ Gr. Nature Publishing Group; 2016; 1–16. doi:10.1038/srep39024

11. Ghorbel M, Cotelle V, Ebel C, Zaidi I, Ormancey M, Galaud J-P, et al. Regulation of the wheat MAP Kinase Phosphatase 1 by 14-3-3 proteins. Plant Sci. Elsevier Ireland Ltd; 2017;257: 37–47. doi:10.1016/j.plantsci.2017.01.006

12. Singh RK, Redoña E, Refuerzo L. Varietal Improvement for Abiotic Stress Tolerance in Crop Plants: Special Reference to Salinity in Rice. In: Pareek A, Sopory SK, Bohnert HJ, editors. Abiotic Stress Adaptation in Plants: Physiological, Molecular and Genomic Fundation. Dordrecht: Springer Netherlands; 2010. pp. 387–415. doi:10.1007/978-90-481-3112-9

13. Huang G-T, Ma S-L, Bai L-P, Zhang L, Ma H, Jia P, et al. Signal transduction during cold, salt, and drought stresses in plants. Mol Biol Rep. 2012;39: 969–987. doi:10.1007/s11033-011-0823-1

14. Xiong L, Zhu J-K. Regulation of Abscisic Acid Biosynthesis. Plant Physiol. 2003;133: 29–36. doi:10.1104/pp.103.025395.mutant

15. Yamaguchi-Shinozaki K, Shinozaki K. Transcriptional Regulatory Networks in Cellular Responses and Tolerance to Dehydration and Cold Stresses. Annu Rev Plant Biol. 2006;57: 781–803. doi:10.1146/annurev.arplant.57.032905.105444

16. Lind C, Dreyer I, López-Sanjurjo EJ, von Meyer K, Ishizaki K, Kohchi T, et al. Stomatal Guard Cells Co-opted an Ancient ABA-Dependent Desiccation Survival System to Regulate Stomatal Closure. Curr Biol. 2015;25: 928–935. doi:10.1016/j.cub.2015.01.067

17. Zingaretti SM, Inácio MC, de Matos Pereira L, Paz TA, de Castro França S. Water Stress and Agriculture. Responses of Organisms to Water Stress. InTech; 2013. pp. 151–179.

18. Padmalatha KV, Dhandapani G, Kanakachari M, Kumar S, Dass A, Patil DP, et al. Genome-wide transcriptomic analysis of cotton under drought stress reveal significant down-regulation of genes and pathways involved in fibre elongation and up-regulation of defense responsive genes. Plant Mol Biol. 2012;78: 223–246. doi:10.1007/s11103-011-9857-y

19. Tognetti VB, Mühlenbock P, van Breusegem F. Stress homeostasis - the redox and auxin perspective. Plant, Cell Environ. 2012;35: 321–333. doi:10.1111/j.1365-3040.2011.02324.x

20. Nobuta K, Okrent RA, Stoutemyer M, Rodibaugh N, Kempema L, Wildermuth MC, et al. The GH3 Acyl Adenylase Family Member PBS3 Regulates Salicylic Acid-Dependent Defense Responses in Arabidopsis. Plant Physiol. 2007;144: 1144–1156. doi:10.1104/pp.107.097691

21. Zhu Y, Li Y, Xin D, Chen W, Shao X, Wang Y, et al. RNA-Seq-based transcriptome analysis of dormant flower buds of Chinese cherry (*Prunus pseudocerasus*). Gene. Elsevier B.V.; 2015;555: 362–376. doi:10.1016/j.gene.2014.11.032

22. Datta R, Kumar D, Sultana A, Hazra S, Bhattacharyya D, Chattopadhyay S. Glutathione regulates ACC synthase transcription via WRKY33 and ACC oxidase by modulating mRNA stability to induce ethylene synthesis during stress. Plant Physiol. 2015;169: pp.01543.2015. doi:10.1104/pp.15.01543

23. Wilkinson S, Davies WJ. ABA-based chemical signalling: The co-ordination of responses to stress in plants. Plant, Cell Environ. 2002;25: 195–210. doi:10.1046/j.0016-8025.2001.00824.x

24. Nishiyama R, Watanabe Y, Fujita Y, Le DT, Kojima M, Werner T, et al. Analysis of Cytokinin Mutants and Regulation of Cytokinin Metabolic Genes Reveals Important Regulatory Roles of Cytokinins in Drought, Salt and Abscisic Acid Responses, and Abscisic Acid Biosynthesis. Plant Cell. 2011;23: 2169–2183. doi:10.1105/tpc.111.087395

25. He W, Zhuang H, Fu Y, Guo L, Guo B, Guo L, et al. *De novo*Transcriptome Assembly of a Chinese Locoweed (*Oxytropis ochrocephala*) Species Provides Insights into Genes Associated with Drought, Salinity, and Cold Tolerance. Front Plant Sci. 2015;6: 1086. doi:10.3389/fpls.2015.01086

26. Pospíšilová H, Jiskrová E, Vojta P, Mrízová K, Kokáš F, Čudejková MM, et al. Transgenic barley overexpressing a cytokinin dehydrogenase gene shows greater tolerance to drought stress. N Biotechnol. 2016;33. doi:10.1016/j.nbt.2015.12.005

27. Zawaski C, Busov VB. Roles of Gibberellin Catabolism and Signaling in Growth and Physiological Response to Drought and Short-Day Photoperiods in *Populus* trees. PLoS One. 2014;9: e86217. doi:10.1371/journal.pone.0086217

28. Zhang Y, Lan H, Shao Q, Wang R, Chen H, Tang H, et al. An A20/AN1-type zinc finger protein modulates gibberellins and abscisic acid contents and increases sensitivity to abiotic stress in rice (*Oryza sativa*). J Exp Bot. 2016;67: 315–326. doi:10.1093/jxb/erv464

29. Chen J-H, Jiang H-W, Hsieh E-J, Chen H-Y, Chien C-T, Hsieh H-L, et al. Drought and salt stress tolerance of an Arabidopsis glutathione S-transferase U17 knockout mutant are attributed to the combined effect of glutathione and abscisic acid. Plant Physiol. 2012;158: 340–51. doi:10.1104/pp.111.181875

30. Bartels D, Sunkar R. Drought and Salt Tolerance in Plants. CRC Crit Rev Plant Sci. 2005;24: 23–58. doi:10.1080/07352680590910410

31. Wang X, Zhang W, Li W, Mishra G. Phospholipid signaling in plant response to drought and salt stress. In: M.A. J, Hasegawa PM, Jain SM, editors. Advances in Molecular Breeding Toward drought and Salt Tolerant Crops. Dordrecht: Springer; 2007. pp. 183–192.

32. Xiong L, Schumaker K, Zhu J-K. Cell Signaling during Cold, Drought, and Salt Stress. Plant Cell. 2002; 165–184. doi:10.1105/tpc.000596.S166

33. Mishra G, Zhang W, Deng F, Zhao J, Wang X. A Bifurcating Pathway Directs Abscisic Acid Effects on Stomatal Closure and Opening in *Arabidopsis*. Science (80- ). 2006;312: 264–266.

34. Du D, Hao R, Cheng T, Pan H, Yang W, Wang J, et al. Genome-Wide Analysis of the *AP2/ERF* Gene Family in *Prunus mume*. Plant Mol Biol Report. 2013;31: 741–750. doi:10.1007/s11105-012-0531-6

35. Fu M, Kang HK, Son SH, Kim SK, Nam KH. A subset of Arabidopsis RAV transcription factors modulates drought and salt stress responses independent of ABA. Plant Cell Physiol. 2014;55: 1892–1904. doi:10.1093/pcp/pcu118

36. Skubacz A, Daszkowska-Golec A, Szarejko I. The Role and Regulation of ABI5 (ABA-Insensitive 5) in Plant Development, Abiotic Stress Responses and Phytohormone Crosstalk. Front Plant Sci. 2016;7: 1–17. doi:10.3389/fpls.2016.01884

37. Mizoi J, Shinozaki K, Yamaguchi-Shinozaki K. AP2/ERF family transcription factors in plant abiotic stress responses. Biochim Biophys Acta. Elsevier B.V.; 2012;1819: 86–96. doi:10.1016/j.bbagrm.2011.08.004

38. Sakuma Y, Maruyama K, Osakabe Y, Quin F, Seki M, Shinozaki K, et al. Functional analysis of an *Arabidopsis* transcription factor, DREB2A, involved in drought-responsive gene expression. Plant Cell. 2006;18: 1292–1309. doi:10.1105/tpc.105.035881.1

39. Sazegari S, Niazi A, Ahmadi SF. A study on the regulatory network with promoter analysis for Arabidopsis *DREB*-genes. Bioinformation. 2015;11: 973–2063. doi:10.6026/97320630011101

40. Jin L-G, Li H, Liu J-Y. Molecular characterization of three ethylene responsive element binding factor genes from cotton. J Integr Plant Biol. 2010;52: 485–495. doi:10.1111/j.1744-7909.2010.00914.x

41. Castilhos G, Lazzarotto F, Spagnolo-Fonini L, Bodanese-Zanettini MH, Margis-Pinheiro M. Possible roles of basic helix-loop-helix transcription factors in adaptation to drought. Plant Sci. Elsevier Ireland Ltd; 2014;223: 1–7. doi:10.1016/j.plantsci.2014.02.010

42. Ariel FD, Manavella PA, Dezar CA, Chan RL. The true story of the HD-Zip family. Trends Plant Sci. 2007;12: 419–426. doi:10.1016/j.tplants.2007.08.003

43. Wang H, Lin J, Li XG, Chang Y. Genome-wide identification of pear HD-Zip gene family and expression patterns under stress induced by drought, salinity, and pathogen. Acta Physiol Plant. Springer Berlin Heidelberg; 2015;37: 1–19. doi:10.1007/s11738-015-1933-5

44. Wei W, Zhang Y-Q, Tao J-J, Chen H-W, Li Q-T, Zhang W-K, et al. The Alfin-like homeodomain finger protein AL5 suppresses multiple negative factors to confer abiotic stress tolerance in Arabidopsis. Plant J. 2015;81: 871–883. doi:10.1111/tpj.12773

45. Cheng S, Zhou D-X, Zhao Y. *WUSCHEL*-related homeobox gene *WOX11* increases rice drought resistance by controlling root hair formation and root system development. Plant Signal Behav. Taylor & Francis; 2016;11: e1130198. doi:10.1080/15592324.2015.1130198

46. Singh D, Laxmi A. Transcriptional regulation of drought response: a tortuous network of transcriptional factors. Front Plant Sci. 2015;6: 895. doi:10.3389/fpls.2015.00895

47. Tripathi P, Rabara RC, Rushton PJ. A systems biology perspective on the role of WRKY transcription factors in drought responses in plants. Planta. 2014;239: 255–266. doi:10.1007/s00425-013-1985-y

48. Olsen AN, Ernst HA, Leggio LL, Skriver K. NAC transcription factors: Structurally distinct, functionally diverse. Trends Plant Sci. 2005;10: 79–87. doi:10.1016/j.tplants.2004.12.010

49. Xu Z, Wang C, Xue F, Zhang H, Ji W. Wheat NAC transcription factor TaNAC29 is involved in response to salt stress. Plant Physiol Biochem. Elsevier Masson SAS; 2015;96: 356–363. doi:10.1016/j.plaphy.2015.08.013

50. Janiak A, Kwas̈niewski M, Szarejko I. Gene expression regulation in roots under drought. J Exp Bot. 2016;67: 1003–1014. doi:10.1093/jxb/erv512

51. Han X, Tang S, An Y, Zheng DC, Xia XL, Yin WL. Overexpression of the poplar *NF-YB7* transcription factor confers drought tolerance and improves water-use efficiency in *Arabidopsis*. J Exp Bot. 2013;64: 4589–4601. doi:10.1093/jxb/ert262

52. Li K-Q, Xu X-Y, Huang X-S. Identification of differentially expressed genes related to dehydration resistance in a highly drought-tolerant pear, *Pyrus betulaefolia*, as through RNA-Seq. PLoS One. 2016;11: e0149352. doi:10.1371/journal.pone.0149352

53. Bhardwaj AR, Joshi G, Kukreja B, Malik V, Arora P, Pandey R, et al. Global insights into high temperature and drought stress regulated genes by RNA-Seq in economically important oilseed crop *Brassica juncea*. BMC Plant Biol. 2015;15: 9. doi:10.1186/s12870-014-0405-1

54. Liu J, Zhang C, Wei C, Liu X, Wang M, Yu F, et al. The RING Finger Ubiquitin E3 Ligase OsHTAS Enhances Heat Tolerance by Promoting H_2_O_2_-Induced Stomatal Closure in Rice. Plant Physiol. 2016;170: 429–443. doi:10.1104/pp.15.00879

55. Rai AC, Singh M, Shah K. Engineering drought tolerant tomato plants over-expressing *BcZAT12* gene encoding a C_2_H_2_ zinc finger transcription factor. Phytochemistry. Elsevier Ltd; 2013;85: 44–50. doi:10.1016/j.phytochem.2012.09.007

56. Vij S, Tyagi AK. Genome-wide analysis of the stress associated protein (SAP) gene family containing A20/AN1 zinc-finger(s) in rice and their phylogenetic relationship with *Arabidopsis*. Mol Genet Genomics. 2006;276: 565–75. doi:10.1007/s00438-006-0165-1

57. Tang S, Liang H, Yan D, Zhao Y, Han X, Carlson JE, et al. *Populus euphratica*: The transcriptomic response to drought stress. Plant Mol Biol. 2013;83: 539–557. doi:10.1007/s11103-013-0107-3

58. Nakashima K, Yamaguchi-Shinozaki K. ABA signaling in stress-response and seed development. Plant Cell Rep. 2013;32: 959–70. doi:10.1007/s00299-013-1418-1

59. Cui F, Brosché M, Lehtonen MT, Amiryousefi A, Xu E, Punkkinen M, et al. Dissecting Abscisic Acid Signaling Pathways Involved in Cuticle Formation. Mol Plant. 2016;9: 926–938. doi:10.1016/j.molp.2016.04.001

60. Buda GJ, Barnes WJ, Fich E a, Park S, Yeats TH, Zhao L, et al. An ATP binding cassette transporter is required for cuticular wax deposition and desiccation tolerance in the moss *Physcomitrella patens*. Plant Cell. 2013;25: 4000–13. doi:10.1105/tpc.113.117648

61. Jin Z, Xue S, Luo Y, Tian B, Fang H, Li H, et al. Hydrogen sulfide interacting with abscisic acid in stomatal regulation responses to drought stress in *Arabidopsis*. Plant Physiol Biochem. Elsevier Masson SAS; 2013;62: 41–46. doi:10.1016/j.plaphy.2012.10.017

62. Brandt B, Brodsky DE, Xue S, Negi J, Iba K, Kangasjarvi J, et al. Reconstitution of abscisic acid activation of SLAC1 anion channel by CPK6 and OST1 kinases and branched ABI1 PP2C phosphatase action. PNAS. 2012;109: 10593–8. doi:10.1073/pnas.1116590109

63. Ksouri N, Jiménez S, Wells CE, Contreras-Moreira B, Gogorcena Y. Transcriptional Responses in Root and Leaf of *Prunus persica* under Drought Stress Using RNA Sequencing. Front Plant Sci. 2016;7: 1–19. doi:10.3389/fpls.2016.01715

64. Wang J, Zheng R, Bai S, Gao X, Liu M, Yan W. Mongolian Almond (*Prunus mongolica* Maxim): The Morpho-Physiological, Biochemical and Transcriptomic Response to Drought Stress. PLoS One. 2015;10: e0124442. doi:10.1371/journal.pone.0124442

65. Liu C, Li C, Liang D, Ma F, Wang S, Wang P, et al. Aquaporin expression in response to water-deficit stress in two *Malus* species: Relationship with physiological status and drought tolerance. Plant Growth Regul. 2013;70: 187–197. doi:10.1007/s10725-013-9791-x

66. Pou A, Medrano H, Flexas J, Tyerman SD. A putative role for TIP and PIP aquaporins in dynamics of leaf hydraulic and stomatal conductances in grapevine under water stress and re-watering. Plant, Cell Environ. 2013;36: 828–843. doi:10.1111/pce.12019

67. Li C, Ng CK-Y, Fan L. MYB transcription factors, active players in abiotic stress signaling. Environ Exp Bot. Elsevier B.V.; 2015;114: 80–91. doi:10.1016/j.envexpbot.2014.06.014

68. Kuromori T, Miyaji T, Yabuuchi H, Shimizu H, Sugimoto E, Kamiya A, et al. ABC transporter AtABCG25 is involved in abscisic acid transport and responses. PNAS. 2010;107: 2361–2366. doi:10.1073/pnas.0912516107

69. Van Houtte H, Vandesteene L, López-Galvis L, Lemmens L, Kissel E, Carpentier S, et al. Overexpression of the trehalase gene *AtTRE1* leads to increased drought stress tolerance in Arabidopsis and is involved in abscisic acid-induced stomatal closure. Plant Physiol. 2013;161: 1158–1171. doi:10.1104/pp.112.211391
