# Supplementary material for: Identification of water use efficiency related genes in ‘Garnem’ almond-peach rootstock using time-course transcriptome analysis
Source: PLoS One. 2018 Oct 11;13(10):e0205493. doi: 10.1371/journal.pone.0205493 (PMC6181374; doi:10.1371/journal.pone.0205493)
Supplement: S3 Appendix — (DOCX) [file pone.0205493.s012.docx]

# S3 Appendix Sections

**Identification of water use efficiency related genes in ‘Garnem’ almond-peach rootstock using time-course transcriptome analysis**

Beatriz Bielsa^1¶^, Seanna Hewitt^2, 3¶^, Sebastian Reyes-Chin-Wo^4^, Amit Dhingra^2, 3*^, María José Rubio-Cabetas^1*^

**^*^Corresponding authors:**

E-mail: adhingra@wsu.edu (AD)

E-mail: mjrubioc@cita-aragon.es (MJR-C)

## S3. Additional mediators of stress signal

**Phytohormone-mediated signal transduction.** Abscisic acid (ABA) is an important phytohormone that accumulates in plant tissues following the onset of drought [13]. Several DEGs representing key genes involved in ABA biosynthesis [3,14,15] were observed to be upregulated at 2 h, including genes encoding *zeaxanthin chloroplastic*, zeaxanthin epoxidase (ZEP), *9-cis*-epoxycarotenoid dioxygenase chloroplastic (NCED) and molybdenum cofactor sulfurase (ABA3) (S3 and S7 Tables). Drought induced accumulation of ABA, followed by ABA-dependent signalling, leads to the synthesis of stress-associated proteins and secondary metabolites. This, in turn stimulates physiological responses such as stomatal closure, consequently increasing tolerance to drought [4,16]. ABA is also involved in crosstalk with other phytohormones that accumulate during drought response, including auxin, ethylene, cytokinin, gibberellic acid, brassinosteroids, salicylic acid (SA), and jasmonic acid (JA) to mediate stress adaptation [17]. While ABA signalling is involved predominantly in stomatal closure and growth cessation responses, auxin signalling, mediated by auxin/indole-3-acetic acid (Aux/IAA) and IAA amido synthetase 3 (GH3), serves to regulate homeostasis as well as ROS signalling and scavenging [18]. Among the genes involved in auxin homeostasis regulation, GH3 is one of the most important with regards to the hormone crosstalk that mediates drought stress adaptation [19,20]. In this study, 3 DEGs encoding putative *GH3* proteins were identified, suggesting that the stress-signalling network is reliant on hormone accumulation and response in ‘Garnem’. In addition to GH3, 4 DEGs encoding small auxin up RNA (SAUR) proteins, 11 DEGs encoding various auxin-induced-like proteins, 3 DEGs encoding auxin-binding proteins (ABPs), 4 DEGs encoding auxin efflux carrier family proteins, and 23 DEGs encoding auxin response factors (ARFs) – all related to auxin signal transduction were found to be upregulated (S3 and S7 Tables) in this study [21]. The observed changes in expression of these genes during drought stress in ‘Garnem’ suggest that regulation of auxin content is involved in the response to drought. Closely linked to auxin biosynthesis and signalling, and therefore also key to drought stress response, is ethylene metabolism [3]. Datta et al. [22] demonstrated that glutathione, a metabolite characteristically produced during stress responses, induces ethylene biosynthesis by modulation of two key enzymes, 1-aminocyclopropane-1-carboxylate synthase (ACS) and 1-aminocyclopropane-1-carboxylate oxidase (ACO). In the present study, a number of DEGs were annotated as ACS and ACO encoding enzymes, indicating that ethylene biosynthesis was activated during drought stress (S7 Table). Also, DEGs representing ethylene-responsive transcription factor (ERF) families, including ERF1, which plays an important role in integrating ethylene and JA-signalling pathways in drought adaptation were also identified [22] (S7 Table). In addition to ABA, auxin, and ethylene, cytokinins, gibberellins, and glutathione-S-transferases are also involved in mediating a response to stress. In non-drought conditions, increased cytokinin concentration in xylem causes a decrease in ABA-induced stomatal closure [23]. Consistent with this finding, Nishiyama et al. [24] demonstrated that a reduction of cytokinin content helps to maintain an elevated water level due to reduced stomatal aperture and protection of the membrane structures. In this study, 17 DEGs encoding laccase enzymes and 13 DEGs encoding cytokinin dehydrogenase-like enzymes (CKXs), both of which are responsible for catalysis of cytokinin degradation, were identified to be upregulated [25,26]. Gibberellin catabolism, mediated by expression of proteins such as GA 20-oxidase (GA20ox), GA30-oxidase (GA30ox) and DELLA, results in decreased growth and increased adaptive response to abiotic stress [27,28]. A total of 4 DEGs encoding GA20ox enzymes, 2 DEGs encoding GA30ox enzymes, and 4 DEGs corresponding to DELLA proteins (including 2 GA-insensitive proteins and 2 GA repressor proteins) demonstrated a higher expression level (S3 and S7 Tables).

The aforementioned hormones, along with brassinosteroids, salicylic acid (SA) and jasmonic acid (JA), induce ROS detoxification enzymes, such as glutathione S-transferases (GSTs). Through maintenance of cell redox homeostasis, stress-induced oxidative damage is minimized, resulting in improved tolerance to drought stress [13,19,29]. A total of 51 DEGs, encoding different members of GST family, including GSTDHAR, GSTF13, GSTF6, GSTL3, GSTT1, GSTU10, GSTU17, GSTU9, GSTZ1, etc. were found to be both up- and downregulated (S3 and S7 Tables). Taken together, these results suggest that adaptive drought responses in ‘Garnem’, as in poplar [27], are regulated by various phytohormones working in concert to reduce water loss through stomata, maintain homeostasis, decrease growth related metabolism, and reduce oxidative damage resulting from drought conditions.

**Phospholipid-mediated signal transduction.** Drought stress results in alteration of plasma membrane phospholipid composition. In such instances, phospholipids act as precursors for the generation of secondary messenger molecules that mediate adaptive responses to abiotic stress [30–32]. Four phospholipases have thus far been identified: phospholipase A1 and A2 (PL A1 and PL A2); phospholipase C (PLC); and phospholipase D (PLD) [30]. Drought stress induces overexpression of phosphoinositide-specific phospholipase C (PI-PLC), leading to production of diacylglycerol (DAG) and inositol 1,4,5-trisphosphate (IP_3_), which act as second messengers [30,32] that function in induction of stomatal closure [30,33]. Only one DEG encoding an upregulated PI-PLC, and multiple DEGs encoding different PL A1, PL A2 and PLD genes were identified to be differentially expressed (S7 Table), suggesting the importance of these proteins in drought tolerance adaptation via induction of stomatal closure mechanisms in ‘Garnem’.

**Transcription factor-mediated signal perpetuation.** As a result of drought-induced signalling cascades, kinases and phosphatases target different transcription factors (TFs) that recognize *cis*-elements in promoter regions of effector genes, subsequently activating gene expression in response to drought stress. In ‘Garnem’, the activation of a large number of DEGs representing various TFs lends support to the importance of transcriptional regulatory elements in maintaining homeostasis during drought stress in *Prunus*. Previous studies have provided evidence for the crucial role of the AP1/ERF TF family comprised of AP2, RAV and ERF groups in stress adaptation [34]. RAV1 and its homolog, RAV2, are negative regulators of ABI5, a bZIP TF that plays a key role in ABA signalling, and thereby plays an indirect, but important, role in mediating water loss through stomata [35,36]. DEGs encoding RAV1 and RAV2, as well as two additional DEGs encoding *ABI5* were found to be upregulated at 2 h time point. The expression of *ABI5* gene was also upregulated at the 24 h time point (S3 and S7 Tables). Notably, a DEG encoding an ABI5 homolog, the bZIP TRAB1-like protein, was upregulated at 2 h (S3 and S7 Tables). These observations suggest that in *Prunus*, RAV TFs may function as negative regulators during ABA signalling. Various studies have indicated that DREB TFs, members of the ERF group of transcription factors, play an important role in enhancing tolerance to multiple abiotic stresses [37–39]. A total of 8 DEGs encoding DREB1B, DREB2A, DREB2B, DREB2D and DREB3 proteins were identified, and their observed upregulation indicates their participation of DREB TFs in drought stress tolerance in *Prunus*. DEGs encoding ERF2, which have been shown to increase in expression during drought stress [40] were also found to be upregulated (S3 and S7 Tables).

DEGs representing the bHLH TFs were also found to be upregulated under drought conditions (S3 and S7 Tables). Members of this TF family regulate stomatal development and cell division/differentiation (SPCH and FAMA), ABA signal transduction (RD22 and MYC2), trichome development and increase of leaf surface boundary layer (GLABRA3) [41]. In ‘Garnem’ roots, DEGs annotated as *SPCH* (1 DEG), *FAMA* (2 DEGs), *MYC2* and *MYC2-like* (6 DEGs) and *GLABRA3* (1 DEG) were identified under drought stress. Furthermore, 57 DEGs encoding bHLH TFs, such as *bHLH84-like*, *bHLH85-like* [41], were also identified.

The HD superfamily was one of the most abundant class of proteins identified, representing 81 annotated DEGs (S7 Table). Changes were also observed in the expression of DEGs encoding *ATHB-12* and *ATHB12-like* (3 DEGs), *ATHB-6-like* (2 DEGs), and *HAT-5-like* (2 DEGs) genes (S3 and S7 Tables), which are induced by drought and implicated in both positive and negative regulation of ABA-dependent signalling [42,43]. One of the important DEGs that is noteworthy is the ALFIN-LIKE 5 PHD finger protein. Previous research has demonstrated that over-expression of *AL5*, a homolog of *ALFIN-LIKE 5*, improves tolerance to drought stress in *Arabidopsis*, thereby inhibiting expression of target genes that function as negative regulators of stress tolerance [44]. The role of ALFIN-LIKE 5 has not previously been implicated in signalling, but has been shown to act as a downstream component [44]. *WOX11*, a TF that has been shown to enhance drought tolerance of the root hairs of rice via developmental modulation [45] was upregulated in this study (S3 and S7 Tables). This suggests that WOX11 might also act as a modulator of the ‘Garnem’ root system under drought conditions. Other important TF families that play a crucial role in response to drought are WRKY, NAC, NF-YB and Myb TFs [46,47]. Each of these TFs display a change in expression in response to drought stress in this study as well (S3 and S7 Tables).

NAC TFs are implicated in shoot meristem development and auxin signalling, as well as in dehydration response [48]. In ‘Garnem’, DEGs encoding a NAC domain-containing 19-like, as well as 3 DEGs encoding NAC29 were observed to be upregulated (S3 and S7 Tables). It is well documented that ANNAC019 binds the *ERD1* (*Early responsive dehydration stress 1*) promoter in response to drought. Furthermore, overexpression of NAC29 has been shown to enhance drought tolerance in wheat [49]. In addition to WRKY and NAC TFs, 70 DEGs in the current dataset were annotated as MYB TFs (S7 Table), 11 of which encode Myb-related Myb4 and Myb4-like proteins. Expression of *Myb4* in the roots is implicated in biosynthesis of lignin, as well as that of solutes, including glucose, sucrose and proline. This leads to a reestablishment and maintenance of osmotic balance during drought stress, consequently conferring improved tolerance [50]. NF-YB TFs confer drought tolerance by enhancing WUE. An enhancement in WUE was observed when *NF-YB7* from poplar was overexpressed in Arabidopsis [51]. In the ‘Garnem’ transcriptome, one DEG annotated as *NF-YB3*, homologous to *PtNF-YB7*, was upregulated at 2 h time point (S3 and S7 Tables). This finding suggests that overexpression of this TF may improve WUE in *Prunus* and, consequently, enhance tolerance to drought.

In addition to the aforementioned TFs, additional DEGs coding for other TF families were identified, whose involvement in drought response has been demonstrated previously. These families include HSFs in *Pyrus betulaefolia* [52], PLATZs in *Brassica juncea* [53], Zinc-Finger superfamily TFs: RING finger TFs, A20/AN1 TFs, ZATs, C3HC4 TFs, CCCH TFs and C3HC3 TFs in rice and tomato [46,54–56], and uspA proteins in *Populus euphratica* [57] (S7 Table). The role of these TFs remains to be characterized in *Prunus*, however, they could serve as additional candidates in the strategy for enhancing drought resistance in *Prunus*.

# References

1. Xiong L, Zhu J-K. Abiotic stress signal transduction in plants: Molecular and genetic perspectives. Physiol Plant. 2001;112: 152–166. doi:10.1034/j.1399-3054.2001.1120202.x

2. Ye Y, Ding Y, Jiang Q, Wang F, Sun J, Zhu C. The role of receptor-like protein kinases (*RLKs*) in abiotic stress response in plants. Plant Cell Rep. Springer Berlin Heidelberg; 2017;36: 235–242. doi:10.1007/s00299-016-2084-x

3. Mahajan S, Tuteja N. Cold, salinity and drought stresses: an overview. Arch Biochem Biophys. 2005;444: 139–58. doi:10.1016/j.abb.2005.10.018

4. Roychoudhury A, Paul S, Basu S. Cross-talk between abscisic acid-dependent and abscisic acid-independent pathways during abiotic stress. Plant Cell Rep. 2013;32: 985–1006. doi:10.1007/s00299-013-1414-5

5. Boudsocq M, Sheen J. Stress Signaling II: Calcium Sensing and Signaling. In: Pareek A, Sopory SK, Bohnert HJ, editors. Abiotic Stress Adaptation in Plants: Physiological, Molecular and Genomic Foundation. Dordrecht: Springer Netherlands; 2010. pp. 75–90. doi:10.1007/978-90-481-3112-9

6. Wilkins K, Matthus E, Swarbreck S, Davies J. Calcium-Mediated Abiotic Stress Signaling in Roots. Front Plant Sci. 2016;7: 1–17. doi:10.3389/fpls.2016.01296

7. Zou J-J, Li X-D, Ratnasekera D, Wang C, Liu W-X, Song L-F, et al. Arabidopsis CALCIUM-DEPENDENT PROTEIN KINASE8 and CATALASE3 Function in Abscisic Acid-Mediated Signaling and H_2_O_2_ Homeostasis in Stomatal Guard Cells under Drought Stress. Plant Cell. 2015;27: 1445–60. doi:10.1105/tpc.15.00144

8. Zou J-J, Wei F-J, Wang C, Wu J-J, Ratnasekera D, Liu W-X, et al. Arabidopsis calcium-dependent protein kinase CPK10 functions in abscisic acid- and Ca^2+^-mediated stomatal regulation in response to drought stress. Plant Physiol. 2010;154: 1232–43. doi:10.1104/pp.110.157545

9. Frank W, Munnik T, Kerkmann K, Salamini F, Bartels D. Water deficit triggers phospholipase D activity in the resurrection plant Craterostigma plantagineum. Plant Cell. 2000;12: 111–123.

10. Wang L, Jin X, Li Q, Wang X, Li Z, Wu X. Comparative Proteomics Reveals that Phosphorylation of β-Carbonic Anhydrase 1 Might be Important for Adaptation to Drought Stress in *Brassica napus*. Nat Publ Gr. Nature Publishing Group; 2016; 1–16. doi:10.1038/srep39024

11. Ghorbel M, Cotelle V, Ebel C, Zaidi I, Ormancey M, Galaud J-P, et al. Regulation of the wheat MAP Kinase Phosphatase 1 by 14-3-3 proteins. Plant Sci. Elsevier Ireland Ltd; 2017;257: 37–47. doi:10.1016/j.plantsci.2017.01.006

12. Singh RK, Redoña E, Refuerzo L. Varietal Improvement for Abiotic Stress Tolerance in Crop Plants: Special Reference to Salinity in Rice. In: Pareek A, Sopory SK, Bohnert HJ, editors. Abiotic Stress Adaptation in Plants: Physiological, Molecular and Genomic Fundation. Dordrecht: Springer Netherlands; 2010. pp. 387–415. doi:10.1007/978-90-481-3112-9

13. Huang G-T, Ma S-L, Bai L-P, Zhang L, Ma H, Jia P, et al. Signal transduction during cold, salt, and drought stresses in plants. Mol Biol Rep. 2012;39: 969–987. doi:10.1007/s11033-011-0823-1

14. Xiong L, Zhu J-K. Regulation of Abscisic Acid Biosynthesis. Plant Physiol. 2003;133: 29–36. doi:10.1104/pp.103.025395.mutant

15. Yamaguchi-Shinozaki K, Shinozaki K. Transcriptional Regulatory Networks in Cellular Responses and Tolerance to Dehydration and Cold Stresses. Annu Rev Plant Biol. 2006;57: 781–803. doi:10.1146/annurev.arplant.57.032905.105444

16. Lind C, Dreyer I, López-Sanjurjo EJ, von Meyer K, Ishizaki K, Kohchi T, et al. Stomatal Guard Cells Co-opted an Ancient ABA-Dependent Desiccation Survival System to Regulate Stomatal Closure. Curr Biol. 2015;25: 928–935. doi:10.1016/j.cub.2015.01.067

17. Zingaretti SM, Inácio MC, de Matos Pereira L, Paz TA, de Castro França S. Water Stress and Agriculture. Responses of Organisms to Water Stress. InTech; 2013. pp. 151–179.

18. Padmalatha KV, Dhandapani G, Kanakachari M, Kumar S, Dass A, Patil DP, et al. Genome-wide transcriptomic analysis of cotton under drought stress reveal significant down-regulation of genes and pathways involved in fibre elongation and up-regulation of defense responsive genes. Plant Mol Biol. 2012;78: 223–246. doi:10.1007/s11103-011-9857-y

19. Tognetti VB, Mühlenbock P, van Breusegem F. Stress homeostasis - the redox and auxin perspective. Plant, Cell Environ. 2012;35: 321–333. doi:10.1111/j.1365-3040.2011.02324.x

20. Nobuta K, Okrent RA, Stoutemyer M, Rodibaugh N, Kempema L, Wildermuth MC, et al. The GH3 Acyl Adenylase Family Member PBS3 Regulates Salicylic Acid-Dependent Defense Responses in Arabidopsis. Plant Physiol. 2007;144: 1144–1156. doi:10.1104/pp.107.097691

21. Zhu Y, Li Y, Xin D, Chen W, Shao X, Wang Y, et al. RNA-Seq-based transcriptome analysis of dormant flower buds of Chinese cherry (*Prunus pseudocerasus*). Gene. Elsevier B.V.; 2015;555: 362–376. doi:10.1016/j.gene.2014.11.032

22. Datta R, Kumar D, Sultana A, Hazra S, Bhattacharyya D, Chattopadhyay S. Glutathione regulates ACC synthase transcription via WRKY33 and ACC oxidase by modulating mRNA stability to induce ethylene synthesis during stress. Plant Physiol. 2015;169: pp.01543.2015. doi:10.1104/pp.15.01543

23. Wilkinson S, Davies WJ. ABA-based chemical signalling: The co-ordination of responses to stress in plants. Plant, Cell Environ. 2002;25: 195–210. doi:10.1046/j.0016-8025.2001.00824.x

24. Nishiyama R, Watanabe Y, Fujita Y, Le DT, Kojima M, Werner T, et al. Analysis of Cytokinin Mutants and Regulation of Cytokinin Metabolic Genes Reveals Important Regulatory Roles of Cytokinins in Drought, Salt and Abscisic Acid Responses, and Abscisic Acid Biosynthesis. Plant Cell. 2011;23: 2169–2183. doi:10.1105/tpc.111.087395

25. He W, Zhuang H, Fu Y, Guo L, Guo B, Guo L, et al. *De novo*Transcriptome Assembly of a Chinese Locoweed (*Oxytropis ochrocephala*) Species Provides Insights into Genes Associated with Drought, Salinity, and Cold Tolerance. Front Plant Sci. 2015;6: 1086. doi:10.3389/fpls.2015.01086

26. Pospíšilová H, Jiskrová E, Vojta P, Mrízová K, Kokáš F, Čudejková MM, et al. Transgenic barley overexpressing a cytokinin dehydrogenase gene shows greater tolerance to drought stress. N Biotechnol. 2016;33. doi:10.1016/j.nbt.2015.12.005

27. Zawaski C, Busov VB. Roles of Gibberellin Catabolism and Signaling in Growth and Physiological Response to Drought and Short-Day Photoperiods in *Populus* trees. PLoS One. 2014;9: e86217. doi:10.1371/journal.pone.0086217

28. Zhang Y, Lan H, Shao Q, Wang R, Chen H, Tang H, et al. An A20/AN1-type zinc finger protein modulates gibberellins and abscisic acid contents and increases sensitivity to abiotic stress in rice (*Oryza sativa*). J Exp Bot. 2016;67: 315–326. doi:10.1093/jxb/erv464

29. Chen J-H, Jiang H-W, Hsieh E-J, Chen H-Y, Chien C-T, Hsieh H-L, et al. Drought and salt stress tolerance of an Arabidopsis glutathione S-transferase U17 knockout mutant are attributed to the combined effect of glutathione and abscisic acid. Plant Physiol. 2012;158: 340–51. doi:10.1104/pp.111.181875

30. Bartels D, Sunkar R. Drought and Salt Tolerance in Plants. CRC Crit Rev Plant Sci. 2005;24: 23–58. doi:10.1080/07352680590910410

31. Wang X, Zhang W, Li W, Mishra G. Phospholipid signaling in plant response to drought and salt stress. In: M.A. J, Hasegawa PM, Jain SM, editors. Advances in Molecular Breeding Toward drought and Salt Tolerant Crops. Dordrecht: Springer; 2007. pp. 183–192.

32. Xiong L, Schumaker K, Zhu J-K. Cell Signaling during Cold, Drought, and Salt Stress. Plant Cell. 2002; 165–184. doi:10.1105/tpc.000596.S166

33. Mishra G, Zhang W, Deng F, Zhao J, Wang X. A Bifurcating Pathway Directs Abscisic Acid Effects on Stomatal Closure and Opening in *Arabidopsis*. Science (80- ). 2006;312: 264–266.

34. Du D, Hao R, Cheng T, Pan H, Yang W, Wang J, et al. Genome-Wide Analysis of the *AP2/ERF* Gene Family in *Prunus mume*. Plant Mol Biol Report. 2013;31: 741–750. doi:10.1007/s11105-012-0531-6

35. Fu M, Kang HK, Son SH, Kim SK, Nam KH. A subset of Arabidopsis RAV transcription factors modulates drought and salt stress responses independent of ABA. Plant Cell Physiol. 2014;55: 1892–1904. doi:10.1093/pcp/pcu118

36. Skubacz A, Daszkowska-Golec A, Szarejko I. The Role and Regulation of ABI5 (ABA-Insensitive 5) in Plant Development, Abiotic Stress Responses and Phytohormone Crosstalk. Front Plant Sci. 2016;7: 1–17. doi:10.3389/fpls.2016.01884

37. Mizoi J, Shinozaki K, Yamaguchi-Shinozaki K. AP2/ERF family transcription factors in plant abiotic stress responses. Biochim Biophys Acta. Elsevier B.V.; 2012;1819: 86–96. doi:10.1016/j.bbagrm.2011.08.004

38. Sakuma Y, Maruyama K, Osakabe Y, Quin F, Seki M, Shinozaki K, et al. Functional analysis of an *Arabidopsis* transcription factor, DREB2A, involved in drought-responsive gene expression. Plant Cell. 2006;18: 1292–1309. doi:10.1105/tpc.105.035881.1

39. Sazegari S, Niazi A, Ahmadi SF. A study on the regulatory network with promoter analysis for Arabidopsis *DREB*-genes. Bioinformation. 2015;11: 973–2063. doi:10.6026/97320630011101

40. Jin L-G, Li H, Liu J-Y. Molecular characterization of three ethylene responsive element binding factor genes from cotton. J Integr Plant Biol. 2010;52: 485–495. doi:10.1111/j.1744-7909.2010.00914.x

41. Castilhos G, Lazzarotto F, Spagnolo-Fonini L, Bodanese-Zanettini MH, Margis-Pinheiro M. Possible roles of basic helix-loop-helix transcription factors in adaptation to drought. Plant Sci. Elsevier Ireland Ltd; 2014;223: 1–7. doi:10.1016/j.plantsci.2014.02.010

42. Ariel FD, Manavella PA, Dezar CA, Chan RL. The true story of the HD-Zip family. Trends Plant Sci. 2007;12: 419–426. doi:10.1016/j.tplants.2007.08.003

43. Wang H, Lin J, Li XG, Chang Y. Genome-wide identification of pear HD-Zip gene family and expression patterns under stress induced by drought, salinity, and pathogen. Acta Physiol Plant. Springer Berlin Heidelberg; 2015;37: 1–19. doi:10.1007/s11738-015-1933-5

44. Wei W, Zhang Y-Q, Tao J-J, Chen H-W, Li Q-T, Zhang W-K, et al. The Alfin-like homeodomain finger protein AL5 suppresses multiple negative factors to confer abiotic stress tolerance in Arabidopsis. Plant J. 2015;81: 871–883. doi:10.1111/tpj.12773

45. Cheng S, Zhou D-X, Zhao Y. *WUSCHEL*-related homeobox gene *WOX11* increases rice drought resistance by controlling root hair formation and root system development. Plant Signal Behav. Taylor & Francis; 2016;11: e1130198. doi:10.1080/15592324.2015.1130198

46. Singh D, Laxmi A. Transcriptional regulation of drought response: a tortuous network of transcriptional factors. Front Plant Sci. 2015;6: 895. doi:10.3389/fpls.2015.00895

47. Tripathi P, Rabara RC, Rushton PJ. A systems biology perspective on the role of WRKY transcription factors in drought responses in plants. Planta. 2014;239: 255–266. doi:10.1007/s00425-013-1985-y

48. Olsen AN, Ernst HA, Leggio LL, Skriver K. NAC transcription factors: Structurally distinct, functionally diverse. Trends Plant Sci. 2005;10: 79–87. doi:10.1016/j.tplants.2004.12.010

49. Xu Z, Wang C, Xue F, Zhang H, Ji W. Wheat NAC transcription factor TaNAC29 is involved in response to salt stress. Plant Physiol Biochem. Elsevier Masson SAS; 2015;96: 356–363. doi:10.1016/j.plaphy.2015.08.013

50. Janiak A, Kwas̈niewski M, Szarejko I. Gene expression regulation in roots under drought. J Exp Bot. 2016;67: 1003–1014. doi:10.1093/jxb/erv512

51. Han X, Tang S, An Y, Zheng DC, Xia XL, Yin WL. Overexpression of the poplar *NF-YB7* transcription factor confers drought tolerance and improves water-use efficiency in *Arabidopsis*. J Exp Bot. 2013;64: 4589–4601. doi:10.1093/jxb/ert262

52. Li K-Q, Xu X-Y, Huang X-S. Identification of differentially expressed genes related to dehydration resistance in a highly drought-tolerant pear, *Pyrus betulaefolia*, as through RNA-Seq. PLoS One. 2016;11: e0149352. doi:10.1371/journal.pone.0149352

53. Bhardwaj AR, Joshi G, Kukreja B, Malik V, Arora P, Pandey R, et al. Global insights into high temperature and drought stress regulated genes by RNA-Seq in economically important oilseed crop *Brassica juncea*. BMC Plant Biol. 2015;15: 9. doi:10.1186/s12870-014-0405-1

54. Liu J, Zhang C, Wei C, Liu X, Wang M, Yu F, et al. The RING Finger Ubiquitin E3 Ligase OsHTAS Enhances Heat Tolerance by Promoting H_2_O_2_-Induced Stomatal Closure in Rice. Plant Physiol. 2016;170: 429–443. doi:10.1104/pp.15.00879

55. Rai AC, Singh M, Shah K. Engineering drought tolerant tomato plants over-expressing *BcZAT12* gene encoding a C_2_H_2_ zinc finger transcription factor. Phytochemistry. Elsevier Ltd; 2013;85: 44–50. doi:10.1016/j.phytochem.2012.09.007

56. Vij S, Tyagi AK. Genome-wide analysis of the stress associated protein (SAP) gene family containing A20/AN1 zinc-finger(s) in rice and their phylogenetic relationship with *Arabidopsis*. Mol Genet Genomics. 2006;276: 565–75. doi:10.1007/s00438-006-0165-1

57. Tang S, Liang H, Yan D, Zhao Y, Han X, Carlson JE, et al. *Populus euphratica*: The transcriptomic response to drought stress. Plant Mol Biol. 2013;83: 539–557. doi:10.1007/s11103-013-0107-3

58. Nakashima K, Yamaguchi-Shinozaki K. ABA signaling in stress-response and seed development. Plant Cell Rep. 2013;32: 959–70. doi:10.1007/s00299-013-1418-1

59. Cui F, Brosché M, Lehtonen MT, Amiryousefi A, Xu E, Punkkinen M, et al. Dissecting Abscisic Acid Signaling Pathways Involved in Cuticle Formation. Mol Plant. 2016;9: 926–938. doi:10.1016/j.molp.2016.04.001

60. Buda GJ, Barnes WJ, Fich E a, Park S, Yeats TH, Zhao L, et al. An ATP binding cassette transporter is required for cuticular wax deposition and desiccation tolerance in the moss *Physcomitrella patens*. Plant Cell. 2013;25: 4000–13. doi:10.1105/tpc.113.117648

61. Jin Z, Xue S, Luo Y, Tian B, Fang H, Li H, et al. Hydrogen sulfide interacting with abscisic acid in stomatal regulation responses to drought stress in *Arabidopsis*. Plant Physiol Biochem. Elsevier Masson SAS; 2013;62: 41–46. doi:10.1016/j.plaphy.2012.10.017

62. Brandt B, Brodsky DE, Xue S, Negi J, Iba K, Kangasjarvi J, et al. Reconstitution of abscisic acid activation of SLAC1 anion channel by CPK6 and OST1 kinases and branched ABI1 PP2C phosphatase action. PNAS. 2012;109: 10593–8. doi:10.1073/pnas.1116590109

63. Ksouri N, Jiménez S, Wells CE, Contreras-Moreira B, Gogorcena Y. Transcriptional Responses in Root and Leaf of *Prunus persica* under Drought Stress Using RNA Sequencing. Front Plant Sci. 2016;7: 1–19. doi:10.3389/fpls.2016.01715

64. Wang J, Zheng R, Bai S, Gao X, Liu M, Yan W. Mongolian Almond (*Prunus mongolica* Maxim): The Morpho-Physiological, Biochemical and Transcriptomic Response to Drought Stress. PLoS One. 2015;10: e0124442. doi:10.1371/journal.pone.0124442

65. Liu C, Li C, Liang D, Ma F, Wang S, Wang P, et al. Aquaporin expression in response to water-deficit stress in two *Malus* species: Relationship with physiological status and drought tolerance. Plant Growth Regul. 2013;70: 187–197. doi:10.1007/s10725-013-9791-x

66. Pou A, Medrano H, Flexas J, Tyerman SD. A putative role for TIP and PIP aquaporins in dynamics of leaf hydraulic and stomatal conductances in grapevine under water stress and re-watering. Plant, Cell Environ. 2013;36: 828–843. doi:10.1111/pce.12019

67. Li C, Ng CK-Y, Fan L. MYB transcription factors, active players in abiotic stress signaling. Environ Exp Bot. Elsevier B.V.; 2015;114: 80–91. doi:10.1016/j.envexpbot.2014.06.014

68. Kuromori T, Miyaji T, Yabuuchi H, Shimizu H, Sugimoto E, Kamiya A, et al. ABC transporter AtABCG25 is involved in abscisic acid transport and responses. PNAS. 2010;107: 2361–2366. doi:10.1073/pnas.0912516107

69. Van Houtte H, Vandesteene L, López-Galvis L, Lemmens L, Kissel E, Carpentier S, et al. Overexpression of the trehalase gene *AtTRE1* leads to increased drought stress tolerance in Arabidopsis and is involved in abscisic acid-induced stomatal closure. Plant Physiol. 2013;161: 1158–1171. doi:10.1104/pp.112.211391
